# Prevalence and Correlation Analysis of Soil-Transmitted Helminths Infections and Treatment Coverage for Preschool and School Aged Children in Kenya: Secondary Analysis of the National School Based Deworming Program Data

**DOI:** 10.3389/fpubh.2021.645522

**Published:** 2021-07-16

**Authors:** Collins Okoyo, Suzy J. Campbell, Mark Minnery, Chrispin Owaga, Nelson Onyango, Graham Medley, Charles Mwandawiro

**Affiliations:** ^1^Eastern and Southern Africa Centre of International Parasite Control, Kenya Medical Research Institute, Nairobi, Kenya; ^2^School of Mathematics, College of Biological and Physical Sciences, University of Nairobi, Nairobi, Kenya; ^3^Deworm the World, Evidence Action, Washington, DC, United States; ^4^Deworm the World, Evidence Action, Nairobi, Kenya; ^5^Faculty of Public Health and Policy, London School of Hygiene and Tropical Medicine, London, United Kingdom

**Keywords:** correlation analysis, soil-transmitted helminths, treatment coverage, school-based deworming, prevalence, Kenya

## Abstract

**Background:** Soil-transmitted helminths (STH) are among the most common parasitic infections globally, disproportionately affecting children. Treatment of STH in Kenya is often targeted at preschool (PSAC) and school aged (SAC) children delivered through annual mass drug administration (MDA) in primary schools. Understanding group-specific prevalence and dynamics between treatment and coverage is critical for continued treatment success. This study aims to provide detailed information on group-specific infection prevalence and relative reductions (RR), and their relationships with treatment coverage over time. Additionally, it aims to quantify the correlation between the observed school level infection prevalence and treatment coverage.

**Methods:** Secondary analysis of existing data collected between 2012 and 2018 by the monitoring and evaluation (M&E) program of the National School-Based Deworming (NSBD) program was used. The M&E program conducted surveys utilizing cross-sectional study design, at four survey time points, in a nationally-representative sample of schoolchildren across counties in Kenya. In each participating school, the program randomly sampled 108 children per school, of both groups. Infection prevalence was estimated using binomial regression, RR in prevalence using multivariable mixed effects model, statistical correlations using structural equation modeling, and change-point-analysis using the binary segmentation algorithm.

**Results:** Overall, STH prevalence for PSAC was 33.7, 20.2, 19.0, and 17.9% during Year 1 (Y1), Year 3 (Y3), Year 5 (Y5), and Year 6 (Y6) surveys, respectively with an overall RR of 46.9% (*p* = 0.001) from Y1 to Y6. Similarly, overall STH prevalence for SAC was 33.6, 18.4, 14.7, and 12.5% during Y1, Y3, Y5, and Y6 surveys, respectively with an overall RR of 62.6% (*p* < 0.001). An overall (all time points) significant but very weak negative correlation was found between treatment coverage and undifferentiated STH prevalence (*r* = −0.144, *p* = 0.002) among PSAC but not in SAC. Further, we observed inter-county heterogeneity variation in infection prevalence, RR, as well as correlations.

**Conclusion:** The analysis showed that after six rounds of MDA, prevalence of STH has significantly declined among both groups of children, however not to a point where it is not a public health problem (below 1%). The analysis, additionally established an overall significant but weak negative correlation between treatment coverage and prevalence, indicating that the current treatment coverage might not be sufficient to drive the overall STH prevalence to below 1%. These findings will allow STH control programs in Kenya to make decisions that will accelerate the attainment of STH elimination as a public health problem.

## Introduction

Soil-transmitted helminths (STH) primarily *Ascaris lumbricoides, Trichuris trichuira*, and the hookworms; *Necator americanus* and *Ancylostoma duodenale* are among the most widespread neglected tropical diseases (NTDs) globally, affecting more than 1.5 billion people each year (1). The current World Health Organization (WHO) guidelines for the control of STH recommend provision of treatment through mass drug administration (MDA) to vulnerable groups, namely, preschool aged children (PSAC), school aged children (SAC), and women of childbearing age (WCA) (2–4). Under the guidelines both infection monitoring and treatment through MDA focus primarily on treatment frequency driven by undifferentiated prevalence for SAC, with MDA recommended for PSAC and WCA in situations where sustainable delivery mechanisms exist (2).

The focus of the current guidelines on SAC is in recognition that the highest burden of infection generally falls among this group, with subsequent risk of detrimental impact on growth (5) and development (6). The WHO also recognizes the substantial burden of infection among PSAC and the need to scale up coverage of MDA to reach 75% among PSAC as well as SAC by 2020 (3, 7, 8). Treatment coverage and resulting prevalence have shown mixed trends globally, with some countries lagging behind (9). Despite this, many countries have made considerable gains in increasing treatment coverage for SAC, however, less than half of endemic countries also target treatment toward PSAC. These countries yield low coverage levels of ~50% with considerable variability of coverage year on year and across their geography (10). Understanding prevalence and coverage, but also how the two interact across key age-groups is key to future deworming efforts.

The Kenyan National School Based Deworming (NSBD) program was launched in 2012 with the aim of reducing the burden of the disease among SAC. The program provides annual MDA to 66 endemic sub-counties spread out across four endemic regions; Western, Nyanza, Rift Valley, and Coast and three regions with minimal risk; Central, Eastern and North Eastern (11, 12). The NSBD program which forms a key component of the country's national response strategy to control STH and schistosome infections, as a public health problem, functions by administering treatment to all SAC including those out of school (in this case PSAC). The program has so far offered consistent and high MDA coverage for the last 6 years, with some variation at the county level (13). The program's impact, both in terms of treatment coverage and infection control, has been consistently monitored through a robust monitoring and evaluation (M&E) program (14). In large part due to the success of the program, national STH infection levels among school children have declined, as reported by the M&E component of the NSBD program (14–17).

Previous studies (14–17) have described the prevalence and intensity of helminth infection using the Kenyan M&E data across seven years of the program, however, undifferentiated between PSAC and SAC. There has also yet to be correlational analysis performed for risk of infection stratified by treatment coverage across counties covered by the NSBD program. Recognizing the importance of understanding levels and drivers of infection differentiated among PSAC and SAC, we combine pre-existing datasets on treatment coverage and infection prevalence to provide detailed trend correlational, and change-point analyses for counties covered by the NSBD program. This is with the aim of better informing STH control programs to make decisions that will accelerate the attainment of elimination of STH as a public health problem in Kenya.

## Materials and Methods

This was a secondary analysis of existing data collected as part of the M&E component of the Kenyan NSBD program. The study utilized secondary data to; differentiate prevalence between PSAC and SAC for the 6 years of the NSBD, then identify any correlations between changes in treatment coverage at the county level and risk of STH infection. Finally, a change point analysis was performed to demonstrate tipping points at which the greatest reductions in prevalence can be seen relative to changes in coverage. Data was drawn from two primary sources; prevalence surveys and MDA coverage monitoring performed as part of the NSBD program. Both datasets and details of analyses performed are described below.

### Prevalence Data

Large scale surveys for STH prevalence have been conducted at Year 1 (Y1), Year 3 (Y3), Year 5 (Y5), and Year 6 (Y6) since the NSBD inception. Detailed design for these surveys are provided in previous studies (14–17). Briefly, all previous surveys utilized a cross-sectional study design in a nationally-representative, stratified, two-stage sample of schoolchildren across counties in Kenya. For Y1, Y3, and Y5 surveys, an average of 200 schools per survey round in 16 counties in four regions; Western, Nyanza, Rift Valley, and Coast were surveyed before treatment to measure national infection levels. However, for the Y6 survey, 100 schools (5 schools per county) were sampled in 20 counties in six regions; Western, Nyanza, Rift Valley, Coast, Eastern and North Eastern.

In each of the sampled schools, 18 children (nine girls and nine boys) were sampled randomly from each of the six classes, including one early childhood development (ECD) class and classes 2–6, using random numbers, for a total of ~108 children per school. During each survey point, the program processed and examined in duplicate single stool samples from each selected child for identification of STH eggs using the Kato-Katz thick smear technique (16, 18).

Specifically for this study, we structured our analysis by two age groups of children as defined by the Kenyan Ministry of Education; PSAC (children aged 2–4 years) and SAC (children aged 5–14 years) to determine the trend in infection prevalence and its correlation with treatment coverage in the two age groups. This kind of age structured analysis will allow comparability of our results to many other age-structured STH modeling work.

The infection prevalence was defined as the number of children tested positive for each infection divided by the total number of children examined in the school. On the other hand, school level infection prevalence was defined as the averaged infection prevalence observed among schoolchildren surveyed at that school (sample size of 108 children). In this analysis, we determined infection prevalence for two age groups separately, being prevalence data for PSAC and SAC during the four survey time points; Y1, Y3, Y5, and Y6 pre-treatment surveys, so as to enable the calculation of the trend in prevalence and relative reduction (RR) over the 6-year period in the modeling. The overall relative reduction in prevalence was defined as the difference in the infection prevalence between Y1 and Y6 surveys.

**Figure 1:**
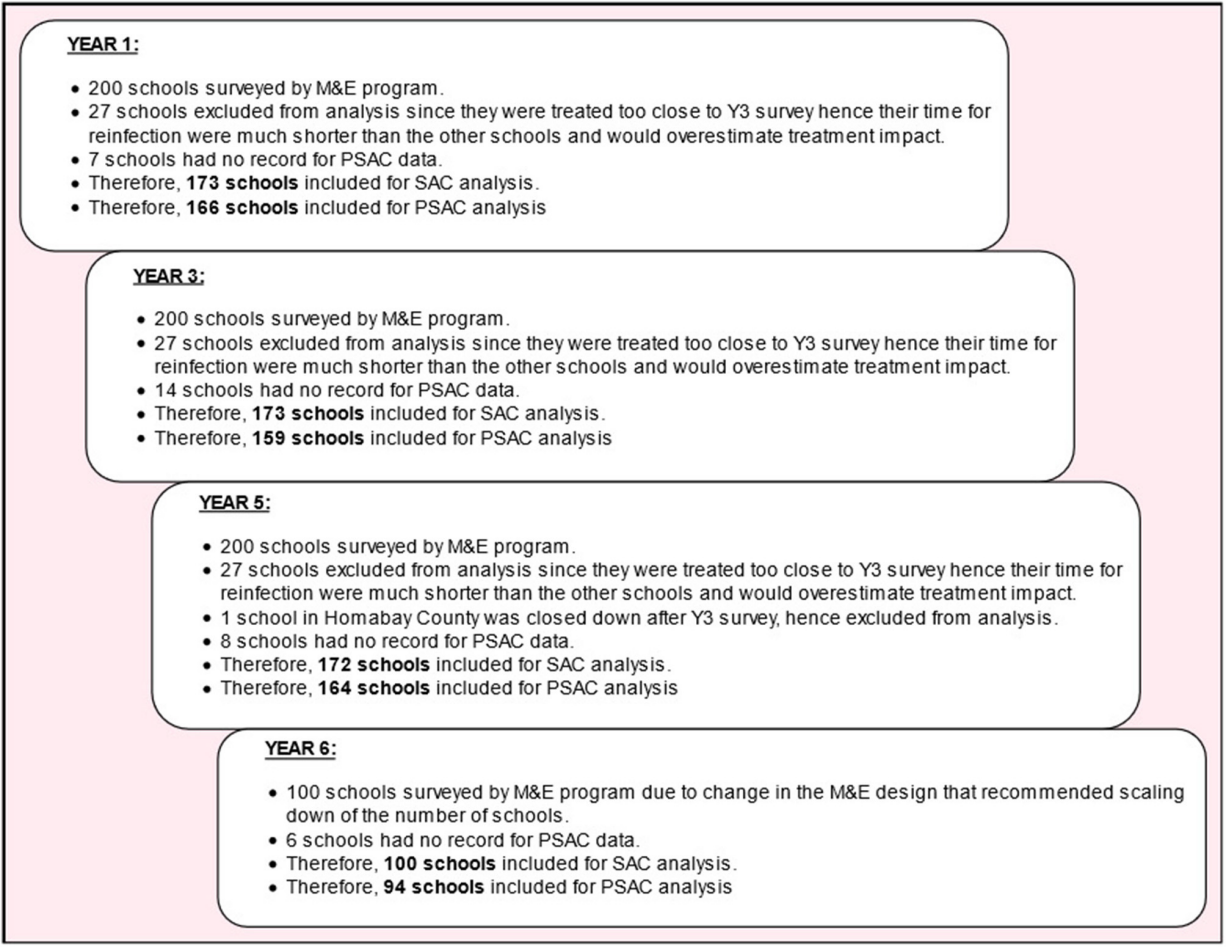
Schema illustrating the number of schools extracted from the NSBDP's M&E database and the number of schools included in this analysis.

Figure 1 shows the number of schools extracted from the NSBDP's M&E database and the number included in this analysis. The M&E program planned to follow the same number of schools (200 schools during Y1 to Y5 and 100 schools during Y6) longitudinally each survey year, however, the following situations led to the final number of schools included in this analysis being inconsistent at some survey points: (1) 27 schools were surveyed too close to Y3 survey hence their time for reinfection was much shorter than the rest of the schools and they were deemed to overestimate the treatment impact. These schools were excluded from the analysis. (2) Some schools had no ECD centers hence they did not have data for PSAC. Such schools were therefore excluded in the analysis for PSAC data. (3) One school was found to be closed down after Y3 survey and was therefore not surveyed during Y5, this school was excluded from the analysis. (4) After Y5 survey, the M&E plan was changed and provided for survey of only 100 schools (five schools per county) during Y6 survey. Taking into account all these situations that led to inconsistent number of schools in some survey years especially among PSAC group and the fact that same number of children were randomly sampled in each school, the analysis objective, which was to determine trend in infection prevalence and its correlation with treatment coverage all aggregated at the county level, was not significantly affected by this inconsistency.

### Treatment Coverage Data

Treatment coverage information for each school is usually recorded within the NSBD program by Evidence Action; an international non-governmental organization, who provides technical support to the government's NSBD program. School-level treatment coverage for each infection were aggregated and presented at the sub-county level. Treatment coverage was determined as the number of children who received treatment for each infection divided by the number targeted at each participating school. Similarly, we calculated the previous year's treatment coverage levels for each of the two age groups separately during the four survey time points with the exception of Y1 survey since there was no previous year treatment. Treatment for STH infections was offered annually to the targeted children using single dose of albendazole in 28 counties in Kenya, consecutively for 6 years. All the 20 counties included in the M&E program were covered for treatment following the WHO guidelines (19). Importantly, treatment was delivered at school level.

### Statistical Analyses

The analysis was conducted for two groups of children (PSAC and SAC) dewormed at the participating schools. The PSAC group of children was defined as those children aged 2–4 years and may not be necessarily enrolled in the school, who were dewormed after presenting themselves at the nearby participating primary school on the deworming day. The SAC group of children was defined as those children aged 5–14 years and enrolled in the primary school, and who were dewormed at their participating primary school on the deworming day.

Infection prevalence, amongst each group of children, was defined as the number of children who were tested positive for a particular STH infection using the Kato-Katz technique by the NSBD program (i.e., $Prevalence={\left(\frac{Number\;of\;children\;positive}{Number\;of\;children\;examined}\right)}^{*}100\%$). For each prevalence estimate calculated, the 95% confidence intervals (CIs) were determined using binomial regression model, taking into account clustering by schools. The relative reductions in STH infection prevalence for each group of children was defined as the difference in prevalence between Y1 and Y6 surveys (i.e., $RR={\left(\frac{Year\;1\;prevalence-Year\;6\;prevalence}{Year\;1\;prevalence}\right)}^{*}$100%) and RR estimates calculated using multivariable mixed effects models with random intercepts for schools and counties, and the associated *p*-values obtained using Wald test. Hence, RR reflected the infection prevalence reduction levels after treatment (in this case, after five rounds of treatment) in comparison with the initial (Y1) prevalence.

Statistical correlations (*r*) between infection prevalence and the previous year's treatment coverage were determined at county-level and the *p*-values estimated using structural equation modeling (SEM), allowing for variations across school clusters. The structure of the SEM model used is shown in Appendix A1. The decision to use SEM over an ordinary correlation model was based on its ability to simultaneously perform statistical tests while handling missing data more elegantly (20, 21). Further, the correlation analysis was stratified by county since each county represents a different transmission pattern and dynamics. Significance levels, for either RR or correlations, were set at *p* ≤ 0.05.

Further, we conducted change point analysis in order to investigate the survey year points associated with significant changes in infection prevalence over the 6 year period, following the yearly MDAs. Briefly, change point analysis helps solve the problem of estimating point(s) at which some statistical properties (e.g., mean) of a time series data show significant change, potentially as a result of an intervention (or lack thereof). More information on the methodology and implementation of this technique is well-illustrated in previous studies (22, 23). In this analysis, we detected significant change point(s) along the 6 year implementation period of the program using the binary segmentation algorithm implementable in the R package “*changepoint*” (24).

Statistical analyses were carried out using either STATA version 14.1 (STATA Corporation, College Station, TX, USA) or R statistical software (25), where appropriate. All graphs were developed using the *ggplot* package implemented in R (26). Maps for school-level prevalence overlaid with their respective subcounty-level treatment coverage were developed using ArcGIS Desktop version 10.2.2 software (Environmental Systems Research Institute Inc., Redlands, CA, USA).

## Results

### Number of Schools and Children Included in the Study

For PSAC group, during Y1, Y3, Y5, and Y6 pre-treatment survey points, children aged 2–4 years were included in the analysis in 166 schools (1,949 children), 159 schools (1,366 children), 164 schools (1,992 children), and 94 schools (781 children), respectively. The number of schools and PSAC children included in the final analysis per county at each survey time point is shown in Table 1. The mean age of PSAC children was 3.7 years (standard deviation 0.5 years) with 51.0% being females.

**Table 1:** Number of schools (children) examined, undifferentiated STH pre-treatment prevalence % (95% CI), and relative reductions % (Wald test: Z-statistic, *p*-value) by county for preschool (PSAC) and school (SAC) aged group of children in 20 counties in Kenya.

**County**	**Number of schools (children) examined**				**Undifferentiated STH pre-treatment prevalence %(95%CI)**				**Relative Reduction (Year 1–Year 6)**
	**Year 1**	**Year 3**	**Year 5**	**Year 6**	**Year 1**	**Year 3**	**Year 5**	**Year 6**	**RR% (Wald test, *p*-value)**
**PSAC Group**									
Bomet	11 (88)	9 (99)	11 (139)	5 (39)	46.6 (30.9–70.1)	31.3 (18.1–54.1)	20.9 (14.6–29.8)	20.5 (13.8–30.4)	56.0 (Z = −3.11, *p* = 0.002)^*^
Bungoma	9 (93)	10 (102)	10 (138)	5 (60)	54.8 (43.5–69.2)	20.6 (12.8–33.1)	15.9 (10.0–25.3)	8.5 (1.9–37.4)	84.5 (Z = −2.56, *p* = 0.011)^*^
Busia	17 (118)	17 (111)	18 (268)	5 (28)	33.1 (22.2–49.2)	26.1 (19.4–35.3)	17.7 (11.4–27.4)	28.6 (10.8–75.5)	13.6 (Z = −0.33, *p* = 0.738)
Garissa	-^ns^	-^ns^	-^ns^	5 (20)	-^ns^	-^ns^	-^ns^	0	-^ns^
Homa Bay	24 (422)	23 (211)	21 (121)	3 (7)	23.9 (18.2–31.5)	17.5 (11.5–26.7)	8.3 (4.0–16.9)	57.1 (24.7–73.2)	*Increase* (38.8%, *p* = 0.031)^**^
Kakamega	19 (177)	18 (175)	19 (254)	5 (42)	44.1 (34.1–56.9)	17.7 (9.2–34.2)	14.9 (9.7–22.7)	26.8 (12.4–58.0)	39.1 (Z = −1.32, *p* = 0.187)
Kericho	12 (161)	10 (96)	12 (166)	5 (37)	38.5 (29.3–50.6)	24.0 (15.6–36.8)	28.5 (19.7–41.3)	21.6 (18.4–25.4)	43.9 (Z = −3.95, *p* < 0.001)^*^
Kilifi	3 (25)	2 (5)	2 (13)	5 (57)	28.0 (18.6–42.1)	20.0 (2.8–32.6)	0	1.8 (0.3–10.7)	93.7 (Z = −2.58, *p* = 0.010)^*^
Kisii	11 (101)	12 (85)	12 (117)	5 (70)	46.5 (36.9–58.6)	34.1 (28.5–40.8)	26.5 (18.8–37.4)	36.2 (24.2–54.3)	22.1 (Z = −1.32, *p* = 0.186)
Kisumu	10 (181)	10 (89)	9 (76)	5 (14)	21.5 (14.9–31.1)	2.2 (0.7–7.3)	2.6 (0.6–12.2)	7.1 (1.2–41.4)	66.8 (Z = −1.34, *p* = 0.181)
Kitui	-^ns^	-^ns^	-^ns^	5 (29)	-^ns^	-^ns^	-^ns^	0	-^ns^
Kwale	9 (98)	10 (52)	10 (191)	5 (33)	25.5 (15.7–41.4)	7.7 (3.4–17.3)	6.4 (3.9–10.3)	12.1 (7.0–21.1)	52.5 (Z = −2.60, *p* = 0.009)^*^
Makueni	-^ns^	-^ns^	-^ns^	4 (12)	-^ns^	-^ns^	-^ns^	0	-^ns^
Migori	8 (127)	8 (83)	8 (78)	5 (66)	18.9 (12.8–27.8)	1.2 (0.2–8.1)	3.9 (0.7–21.9)	4.5 (2.5–8.2)	75.9 (Z = −3.64, *p* < 0.001)^*^
Mombasa	3 (41)	2 (19)	3 (42)	5 (33)	14.6 (3.7–58.6)	10.5 (7.7–14.3)	0	3.0 (0.5–17.6)	79.3 (Z = −1.53, *p* = 0.127)
Narok	10 (99)	7 (75)	9 (83)	5 (61)	55.6 (43.5–70.9)	36.0 (21.1–61.3)	45.8 (31.2–67.2)	37.7 (19.2–74.1)	32.1 (Z = −1.19, *p* = 0.235)
Nyamira	9 (101)	9 (67)	9 (99)	4 (63)	38.6 (27.9–53.4)	29.9 (19.8–45.0)	30.3 (23.1–39.8)	45.2 (35.4–57.6)	*Increase* (17.0%, *p* = 0.338)
Taita Taveta	3 (32)	3 (32)	3 (34)	5 (72)	9.4 (2.0–43.3)	0	2.9 (0.3–33.4)	0	100 (Z = −3.03, *p* = 0.002)^*^
Vihiga	8 (85)	8 (65)	8 (151)	5 (31)	47.1 (39.8–55.6)	27.7 (20.3–37.7)	41.5 (26.3–65.4)	29.0 (16.9–49.8)	38.3 (Z = −1.73, *p* = 0.084)
Wajir	-^ns^	-^ns^	-^ns^	3 (7)	-^ns^	-^ns^	-^ns^	0	-^ns^
**Overall**	**166 (1,949)**	**159 (1,366)**	**164 (1,992)**	**94 (781)**	**33.7 (30.4–37.4)**	**20.2 (17.1–23.9)**	**19.0 (15.9–22.6)**	**17.9 (13.4–23.9)**	**46.9 (Z** **=** **−4.44**, ***p*** **=** 0.001)^*^
**SAC Group**									
Bomet	12 (1,177)	12 (1,185)	12 (1,132)	5 (499)	29.1 (19.7–42.9)	22.8 (15.0–34.6)	17.7 (11.0–28.5)	24.2 (16.6–35.2)	16.7 (Z = −0.71, *p* = 0.476)
Bungoma	10 (921)	10 (920)	10 (895)	5 (456)	48.6 (41.4–57.2)	9.1 (7.2–11.5)	6.1 (3.9–9.6)	4.7 (2.5–8.8)	90.3 (Z = −8.16, *p* < 0.001)^*^
Busia	18 (1,757)	18 (1,780)	18 (1,641)	5 (497)	36.3 (31.6–41.6)	26.0 (19.3–35.0)	16.8 (12.1–23.4)	23.4 (9.0–61.0)	35.5 (Z = −1.04, *p* = 0.297)
Garissa	-^ns^	-^ns^	-^ns^	5 (177)	-^ns^	-^ns^	-^ns^	0	-^ns^
Homa Bay	24 (2,100)	24 (2,238)	23 (2,300)	5 (527)	31.2 (25.6–38.2)	16.3 (11.4–23.4)	11.7 (7.6–18.0)	22.6 (17.7–28.9)	27.6 (Z = −2.27, *p* = 0.023)^*^
Kakamega	20 (1,885)	20 (1,869)	20 (1,801)	5 (490)	29.7 (24.3–36.4)	15.6 (10.9–22.5)	9.1 (5.9–14.0)	23.8 (17.3–32.8)	19.8 (Z = −1.26, *p* = 0.206)
Kericho	12 (1,107)	12 (1,189)	12 (1,094)	5 (498)	28.1 (20.1–39.3)	16.1 (10.9–22.5)	20.0 (13.3–30.1)	16.8 (13.2–21.4)	40.3 (Z = −2.87, *p* = 0.004)^*^
Kilifi	3 (279)	3 (282)	3 (280)	5 (444)	33.3 (31.1–35.7)	5.0 (3.3–7.4)	2.2 (1.4–3.4)	5.3 (1.4–19.6)	84.2 (Z = −2.78, *p* = 0.005)^*^
Kisii	12 (1,178)	12 (1,171)	12 (1,141)	5 (456)	46.9 (40.6–54.1)	25.6 (19.5–33.6)	23.6 (17.7–31.4)	19.6 (12.4–31.1)	58.1 (Z = −4.86, *p* < 0.001)^*^
Kisumu	10 (887)	10 (927)	10 (984)	5 (524)	16.6 (11.9–23.2)	5.0 (3.3–7.4)	4.1 (2.8–6.1)	3.1 (1.2–7.8)	81.5 (Z = −3.68, *p* < 0.001)^*^
Kitui	-^ns^	-^ns^	-^ns^	5 (509)	-^ns^	-^ns^	-^ns^	0.4 (0.1–1.3)	-^ns^
Kwale	10 (912)	10 (917)	10 (813)	5 (485)	29.5 (22.3–39.1)	16.0 (10.1–25.3)	4.4 (2.7–7.1)	5.9 (3.4–10.3)	79.9 (Z = −5.80, *p* < 0.001)^*^
Makueni	-^ns^	-^ns^	-^ns^	5 (510)	-^ns^	-^ns^	-^ns^	0.6 (0.2–2.1)	-^ns^
Migori	8 (718)	8 (773)	8 (736)	5 (467)	23.0 (18.3–28.9)	2.2 (1.5–3.3)	1.9 (1.0–3.7)	2.2 (1.1–4.3)	90.6 (Z = −6.38, *p* < 0.001)^*^
Mombasa	3 (279)	3 (281)	3 (265)	5 (485)	20.8 (10.4–41.5)	2.5 (0.9–7.1)	1.8 (0.2–17.7)	2.2 (0.7–6.4)	89.4 (Z = −3.57, *p* < 0.001)^*^
Narok	10 (932)	10 (966)	10 (951)	5 (442)	53.0 (47.4–59.2)	40.2 (33.1–48.8)	43.3 (36.2–51.7)	23.1 (16.9–31.6)	56.4 (Z = −6.31, *p* < 0.001)^*^
Nyamira	10 (965)	10 (1,000)	10 (956)	5 (448)	30.8 (23.1–41.0)	18.5 (13.6–25.1)	16.4 (12.1–22.2)	19.7 (10.6–36.7)	35.9 (Z = −2.22, *p* = 0.027)^*^
Taita Taveta	3 (285)	3 (281)	3 (281)	5 (417)	2.1 (0.7–5.9)	0	0	0.2 (0–1.7)	88.6 (Z = −1.87, *p* = 0.061)
Vihiga	8 (752)	8 (749)	8 (703)	5 (500)	50.5 (43.4–58.8)	36.7 (27.0–50.0)	31.0 (19.3–49.7)	30.8 (19.1–49.7)	39.0 (Z = −2.36, *p* = 0.018)^*^
Wajir	-^ns^	-^ns^	-^ns^	5 (105)	-^ns^	-^ns^	-^ns^	0	-^ns^
**Overall**	**173 (16,134)**	**173 (16,528)**	**172 (15,973)**	**100 (8,936)**	**33.6 (31.2–36.1)**	**18.4 (16.2–20.9)**	**14.7 (12.6–17.1)**	**12.5 (10.0–15.6)**	**62.6 (Z** **=** **−9.52**, ***p*** **<** 0.001)^*^

*-^n^ Indicates counties which had not had routine parasitological monitoring, hence, surveys were not conducted in these counties during Year 1, Year 3, and Year 5 assessments*.

* *Indicates statistically significant relative reductions since Year 1 (noting that this could be a reflection of sampling technique, and noting also that oftentimes reductions have not been sustained)*.

** *Indicates statistically significant increase in prevalence since Year 1*.

*Data Source: Infection prevalence data are collected and compiled by the Kenya Medical Research Institute (KEMRI), who conducts the monitoring and evaluation of the national school based deworming program*.

For SAC group, during Y1, Y3, Y5, and Y6 pre-treatment survey points, children aged 5–14 years were included in the analysis in 173 schools (16,134 children), 173 schools (16,528 children), 172 schools (15,973 children), and 100 schools (8,936 children), respectively. The number of schools and SAC children included in the final analysis per county at each time point is shown in Table 1. The mean age of SAC children was 10.1 years (standard deviation 2.1 years) with 50.2% being females.

### Treatment Coverage

Overall treatment coverage for PSAC, for each annual MDA from Y1 through to Y6 was 21.6, 82.4, 91.9, 87.5, 75.0, and 84.8%, respectively. Treatment coverage varied within counties across different years of MDA. Across all years there were counties that did not reach WHO recommended MDA coverage of ≥75% coverage (4, 27). In Y1 no county reached ≥75%, in Y2 seven counties, in Y3 one county, in Y4 three counties, in Y5 five counties, and in Y6 five counties. County-level summary of the PSAC STH treatment coverage is outlined in Table 2.

**Table 2:** County level summary of STH treatment coverage (%) for preschool (PSAC) and school (SAC) aged group of children in 20 counties in Kenya.

**County**	**Year 1 (Y1)**			**Year 2 (Y2)**			**Year 3 (Y3)**			**Year 4 (Y4)**			**Year 5 (Y5)**			**Year 6 (Y6)**		
	**Children targeted**	**Children treated**	**Coverage (%)**	**Children targeted**	**Children treated**	**Coverage (%)**	**Children targeted**	**Children treated**	**Coverage (%)**	**Children targeted**	**Children treated**	**Coverage (%)**	**Children targeted**	**Children treated**	**Coverage (%)**	**Children targeted**	**Children treated**	**Coverage (%)**
**PSAC Group**																		
Bomet	361,835	90,532	25.0	72,436	66,889	92.3	78,921	75,191	95.3	83,111	66,978	80.6	71,459	62,122	86.9	71,453	61,209	85.7
Bungoma	574,830	142,113	24.7	146,748	144,725	98.6	169,706	167,233	98.5	204,242	174,960	85.7	196,734	155,710	79.1	184,476	163,125	88.4
Busia	304,959	81,130	26.6	9,7734	87,608	89.6	97,285	90,042	92.6	92,496	86,196	93.2	96,068	86,479	90.0	85,669	79,184	92.4
Garissa	-^nt^	-^nt^	-^nt^	11,674	5,442	46.6	-^nt^	-^nt^	-^nt^	0	3,410	100	0	282	100	0	1,304	100
Homa Bay	390,599	116,100	29.7	122,890	111,517	90.7	115,687	117,934	101.9	115,425	115,130	99.7	127,874	112,808	88.2	117,224	112,872	96.3
Kakamega	655,996	159,125	24.3	223,596	164,303	73.5	213,334	171,309	80.3	227,888	156,299	68.6	231,328	153,966	66.6	270,597	172,142	63.6
Kericho	219,434	55,951	25.5	66,072	58,417	88.4	68,827	70,042	101.8	73,250	65,515	89.4	70,803	68,198	96.3	72,754	68,361	94.0
Kilifi	386,498	107,647	27.9	152,029	111,083	73.1	148,415	114,997	77.5	133,653	116,830	87.4	142,828	63,434	44.4	113,519	59,554	52.5
Kisii	485,344	154,395	31.8	129,635	135,292	104.4	115,767	147,113	127.1	119,848	140,307	117.1	126,756	135,406	106.8	107,293	133,155	124.1
Kisumu	325,997	81,232	24.9	135,127	80,416	59.5	111,586	84,986	76.2	115,260	74,961	65.0	121,750	74,106	60.9	155,070	78,981	50.9
Kitui	-^nt^	-^nt^	-^nt^	6,641	7,426	111.8	-^nt^	-^nt^	-^nt^	0	1,461	100	0	37	100	0	199	100
Kwale	222,952	57,224	25.7	78,543	56,885	72.4	72,943	68,997	94.6	72,846	65,519	89.9	-^nt^	-^nt^	-^nt^	45,124	9,089	20.1
Makueni	-^nt^	-^nt^	-^nt^	19,518	14,713	75.4	-^nt^	-^nt^	-^nt^	0	10,625	100	-^nt^	-^nt^	-^nt^	0	847	100
Migori	387,226	122,454	31.6	124,687	112,646	90.3	121,587	109,443	90.0	121,832	109,808	90.1	129,346	111,460	86.2	140,927	126,102	89.5
Mombasa	171,418	44,979	26.2	82,343	53,718	65.2	75,798	55,024	72.6	77,275	67,931	87.9	91,886	63,589	69.2	93,058	74,882	80.5
Narok	118,162	38,804	32.8	35,012	27,921	79.7	36,256	32,923	90.8	35,500	31,249	88.0	41,334	32,490	78.6	34,775	32,666	93.9
Nyamira	164,404	48,828	29.7	59,437	57,408	96.6	54,120	63,910	118.1	61,591	58,937	95.7	63,272	56,670	89.6	57,215	55,698	97.3
T. Taveta	81,081	18,208	22.5	23,244	19,074	82.1	24,752	22,441	90.7	23,014	23,848	103.6	26,530	23,220	87.5	20,243	19,892	98.3
Vihiga	205,795	45,988	22.3	51,791	44,419	85.8	65,090	50,714	77.9	62,954	45,096	71.6	66,890	46,254	69.1	68,776	46,527	67.7
Wajir	-^nt^	-^nt^	-^nt^	13,661	2,608	19.1	-^nt^	-^nt^	-^nt^	0	1,948	100	0	70	100	0	1,317	100
**Overall**	**5,056,530**	**1,364,710**	**21.6**	**1,652,818**	**1,362,510**	**82.4**	**1,570,074**	**1,442,299**	**91.9**	**1,620,185**	**1417,008**	**87.5**	**1,604,858**	**1,246,301**	**75.0**	**1,638,173**	**1,297,106**	**84.8**
**SAC Group**																		
Bomet	426,116	361,835	84.9	358,918	292,936	81.6	341,674	304,609	89.2	377,363	300,454	79.6	333,515	288,149	86.4	312,436	289,645	92.7
Bungoma	766,796	574,830	75.0	777,099	592,771	76.3	787,615	639,544	81.2	894,388	678,926	75.9	785,497	610,156	77.7	714,089	634,390	88.8
Busia	382,859	304,959	79.7	409,262	317,705	77.6	395,103	328,738	83.2	410,675	331,658	80.8	370,401	329,959	89.1	336,662	323,716	96.2
Garissa	-^nt^	-^nt^	-^nt^	30,753	19,103	62.1	-^nt^	-^nt^	-^nt^	16,280	12,421	76.3	2,391	869	36.3	12,509	10,621	84.9
Homa Bay	558,461	390,599	69.9	550,726	393,454	71.4	532,667	402,400	75.5	536,013	408,226	76.2	489,698	412,220	84.2	477,135	421,617	88.4
Kakamega	823,239	655,996	79.7	832,176	662,831	79.7	811,909	684,524	84.3	859,097	688,322	80.1	854,928	679,122	79.4	778,803	720,725	92.5
Kericho	261,274	219,434	84.0	355,599	270,531	76.1	333,436	292,384	87.7	359,788	296,361	82.4	329,547	297,105	90.2	312,472	297,698	95.3
Kilifi	467,662	386,498	82.6	528,382	387,276	73.3	509,419	388,605	76.3	483,697	395,238	81.7	507,925	348,150	68.5	458,002	377,011	82.3
Kisii	629,268	485,344	77.1	584,798	448,239	76.6	526,545	466,241	88.5	570,673	483,819	84.8	518,861	475,058	91.6	505,358	467,867	92.6
Kisumu	425,619	325,997	76.6	432,260	316,014	73.1	397,356	323,971	81.5	404,793	325,678	80.5	432,915	325,620	75.2	416,778	342,801	82.3
Kitui	-^nt^	-^nt^	-^nt^	34,900	31,756	91.0	-^nt^	-^nt^	-^nt^	8,954	6,480	72.4	354	256	72.3	1,640	1,413	86.2
Kwale	267,093	222,952	83.5	296,727	209,964	70.8	280,807	228,882	81.5	297,693	224,345	75.4	-^nt^	-^nt^	-^nt^	247,719	188,017	75.9
Makueni	-^nt^	-^nt^	-^nt^	94,864	82,612	87.1	-^nt^	-^nt^	-^nt^	58,489	55,832	95.5	353	343	97.2	4,844	5,462	112.8
Migori	473,409	387,226	81.8	514,716	374,144	72.7	499,289	387,415	77.6	538,122	401,460	74.6	508,415	422,242	83.1	503,112	454,767	90.4
Mombasa	241,483	171,418	71.0	264,010	189,225	71.7	263,813	192,264	72.9	278,269	226,744	81.5	377,693	221,725	58.7	297,377	260,431	87.6
Narok	134,409	118,162	87.9	135,059	106,365	78.8	128,961	114,564	88.8	140,531	119,332	84.9	158,419	122,676	77.4	135,281	123,562	91.3
Nyamira	220,445	164,404	74.6	272,814	209,462	76.8	257,987	216,079	83.8	282,951	219,063	77.4	255,927	209,144	81.7	228,431	209,479	91.7
T. Taveta	98,049	81,081	82.7	106,196	84,470	79.5	103,707	89,060	85.8	104,859	91,374	87.1	96,302	88,555	92.0	88,535	86,890	98.1
Vihiga	255,208	205,795	80.6	251,233	211,009	84.0	244,376	211,636	86.6	251,061	205,924	82.0	249,379	209,150	83.9	226,788	184,424	81.3
Wajir	-^nt^	-^nt^	-^nt^	18,552	11,086	59.8	-^nt^	-^nt^	-^nt^	15,059	9,704	64.4	2,128	388	18.2	8,069	7,497	92.9
**Overall**	**6,431,390**	**5,056,530**	**78.6**	**6,493,445**	**5,210,953**	**80.2**	**6,414,664**	**5,270,916**	**82.2**	**6,888,755**	**5,481,361**	**79.6**	**6,274,648**	**5,040,887**	**80.3**	**6,066,040**	**5,408,033**	**89.2**

*-^nt^Indicates counties which did not receive treatment during the respective years*.

*Data Source: Treatment coverage data are collected and compiled by the Evidence Action, an international non-governmental organization who provides technical support to the national school based deworming program. These are the official government-owned and reported treatment coverage data for program*.

For SAC, the overall STH treatment coverage for each annual MDA from Y1 through Y6 was high, at 78.6, 80.2, 82.2, 79.6, 80.3, and 89.2%, respectively. During Y1 MDA, three counties did not achieve treatment coverage of ≥75%, in Y2 eight counties, in Y3 one county, in Y4 three counties, in Y5 five counties, and in Y6 all counties achieved ≥75% coverage (Table 2).

### PSAC Infection Prevalence

**Figure 2:**
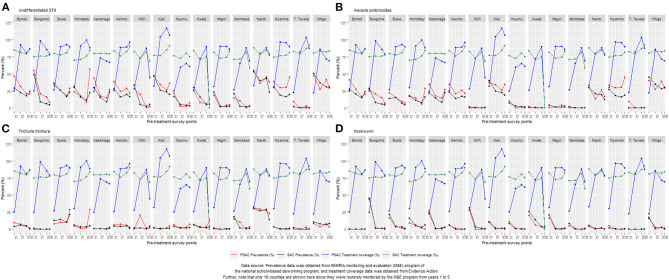
County-level trend of STH infections prevalence and treatment coverage for year 1 (Y1), year 3 (Y3), year 5 (Y5), and year 6 (Y6) survey time points among preschool PSAC) and school (SAC) aged group of children in Kenya. The overlay of undifferentiated STH prevalence with treatment coverage is shown in **(A)**, *Ascaris lumbricoides* **(B)**, *Trichuris trichiura* **(C)**, and hookworm **(D)**.

Overall, 33.7% (95% CI: 30.4–37.4%), 20.2% (95% CI: 17.1–23.9%), 19.0% (95% CI: 15.9–22.6%), and 17.9% (95% CI: 13.4–23.9%) of PSAC children were infected with at least one STH species during Y1, Y3, Y5, and Y6 surveys, respectively, with an overall relative reduction of 46.9% (*p* = 0.001) from Y1 to Y6 (Table 1). The PSAC prevalence of undifferentiated STH differed within counties with highest prevalence, during Y6, observed in Homabay (57.1%) followed by Nyamira (45.2%), Narok (37.7%) and Kisii (36.2%) and zero prevalence observed in Garissa, Kitui, Makueni, Taita Taveta, and Wajir counties. County level relative reduction indicated that only two counties; Kilifi and Taita Taveta, reduced the PSAC prevalence by over 90% (Table 1). Comparison of the county-level trends in PSAC pre-treatment undifferentiated STH prevalence and treatment coverage is outlined in Figure 2A.

*Ascaris lumbricoides* was the most prevalent STH species among PSAC during all four survey time points; Y1, 22.5% (95% CI: 19.3–26.3); Y3, 16.4% (95% CI: 13.5–19.9); Y5, 15.3% (95% CI: 12.5–18.8); and Y6, 13.9% (95% CI: 10.0–19.4), with a statistically significant overall relative reduction (RR = 38.2%, *p* = 0.003). Counties showed differing levels of *A. lumbricoides* prevalence. At Y6, the counties with highest *A. lumbricoides* prevalence among PSAC, of over 20% were; Nyamira (45.2%), Kisii (34.8%), Vihiga (29.0%), Homabay (28.6%), Kakamega (26.8%), Kericho (21.6%), and Bomet (20.5%). Three counties; Kisumu, Kwale and Taita Taveta, showed a statistically significant relative reduction in *A. lumbricoides* prevalence of over 90% (Table 3). Comparison of the county-level trends in PSAC pre-treatment *A. lumbricoides* prevalence and treatment coverage is outlined in Figure 2B.

**Table 3:** Preschool (PSAC) and school (SAC) aged pre-treatment prevalence % (95% CI) and relative reductions % (Wald test: Z-statistic, *p*-value) for *Ascaris lumbricoides* infection in 20 counties in Kenya.

**County**	**Year 1**		**Year 3**		**Year 5**		**Year 6**		**Relative Reduction (Year 1–Year 6) RR% (Wald test**, ***p*****-value)**	
	**PSAC**	**SAC**	**PSAC**	**SAC**	**PSAC**	**SAC**	**PSAC**	**SAC**	**PSAC**	**SAC**
Bomet	40.9 (26.0–64.5)	27.5 (18.6–40.7)	27.3 (14.7–50.6)	20.4 (13.1–31.9)	16.5 (9.7–28.1)	15.0 (8.7–25.9)	20.5 (13.8–30.4)	23.6 (15.9–35.1)	49.9 (Z = −2.39, *p* = 0.017)^*^	14.3 (Z = −0.59, *p* = 0.553)
Bungoma	26.9 (14.6–49.4)	28.6 (19.9–41.0)	20.6 (12.8–33.1)	7.9 (6.5–9.6)	15.2 (9.3–24.8)	5.4 (3.2–9.0)	6.8 (1.7–27.6)	4.7 (2.5–8.8)	74.8 (Z = −1.89, *p* = 0.058)	83.5 (Z = −6.40, *p* < 0.001)^*^
Busia	22.0 (12.2–39.9)	14.1 (10.2–19.3)	15.3 (9.9–23.8)	15.4 (12.1–19.6)	9.6 (5.3–17.5)	6.8 (4.9–9.5)	7.1 (2.7–18.9)	4.6 (1.7–12.3)	67.6 (Z = −2.15, *p* = 0.032)^*^	67.0 (Z = −2.40, *p* = 0.017)^*^
Garissa	-^ns^	-^ns^	-^ns^	-^ns^	-^ns^	-^ns^	0	0	-^ns^	-^ns^
Homa Bay	18.0 (12.5–26.0)	17.2 (12.0–24.8)	14.2 (8.7–23.1)	11.2 (6.9–18.4)	5.8 (2.5–13.5)	7.9 (4.5–13.9)	28.6 (12.3–66.2)	16.3 (10.4–25.8)	*Increase* (58.6%, *p* = 0.264)	5.2 (Z = −0.22, *p* = 0.825)
Kakamega	34.5 (25.2–47.1)	21.8 (16.9–27.9)	17.7 (9.2–34.2)	14.6 (9.9–21.6)	14.5 (9.4–22.3)	8.6 (5.5–13.5)	26.8 (12.4–58.0)	22.6 (15.8–32.4)	22.2 (Z = −0.72, *p* = 0.473)	*Increase* (3.8%, *p* = 0.848)
Kericho	34.8 (25.9–46.8)	23.2 (15.6–34.6)	20.8 (13.8–31.5)	14.1 (9.2–21.8)	26.1 (17.9–38.0)	16.9 (10.8–26.7)	21.6 (18.4–25.4)	15.8 (12.3–20.1)	37.8 (Z = −2.91, *p* = 0.004)^*^	32.1 (Z = −1.89, *p* = 0.059)
Kilifi	0	1.1 (0.4–3.2)	0	0.7 (0.1–4.8)	0	0.4 (0.1–2.5)	0	0.5 (0.1–1.5)	0	57.4 (Z = −0.99, *p* = 0.323)
Kisii	37.6 (26.3–53.9)	39.9 (32.2–49.4)	34.1 (28.5–40.8)	24.7 (18.6–32.8)	25.6 (18.5–35.6)	22.7 (16.8–30.5)	34.8 (24.2–49.9)	19.0 (11.7–30.7)	7.6 (Z = −0.34, *p* = 0.737)	52.4 (Z = −3.76, *p* < 0.001)^*^
Kisumu	9.9 (5.5–18.0)	7.3 (4.7–11.4)	0	2.7 (1.7–4.4)	1.3 (0.1–11.6)	1.7 (1.0–3.0)	0	1.1 (0.6–2.1)	100 (Z = −8.18, *p* < 0.001)^*^	84.3 (Z = −6.26, *p* < 0.001)^*^
Kitui	-^ns^	-^ns^	-^ns^	-^ns^	-^ns^	-^ns^	0	0	-^ns^	-^ns^
Kwale	2.0 (0.3–15.4)	0.5 (0.2–1.3)	0	0.5 (0.2–1.8)	0	0	0	0	100 (Z = −6.58, *p* < 0.001)^*^	100 (Z = −11.62, *p* < 0.001)^*^
Makueni	-^ns^	-^ns^	-^ns^	-^ns^	-^ns^	-^ns^	0	0	-^ns^	-^ns^
Migori	0.8 (0.1–5.4)	3.9 (2.1–7.3)	1.2 (0.2–8.1)	1.4 (0.8–2.6)	3.9 (0.7–21.9)	1.4 (0.6–3.2)	3.0 (1.1–8.2)	1.1 (0.5–2.6)	*Increase* (284.8%, *p* = 0.246)	72.3 (Z = −2.47, *p* = 0.013)^*^
Mombasa	0	1.8 (1.2–2.6)	0	0	0	0	0	0.4 (0.1–3.6)	0	75.5 (Z = −1.22, *p* = 0.223)
Narok	30.3 (17.5–52.6)	29.7 (20.8–42.5)	13.3 (4.8–36.7)	20.9 (15.6–28.1)	26.5 (13.8–50.8)	19.2 (10.9–33.7)	16.4 (4.1–66.3)	12.0 (5.9–24.6)	45.9 (Z = −1.09, *p* = 0.277)	59.6 (Z = −3.46, *p* = 0.001)^*^
Nyamira	32.7 (21.0–50.8)	26.9 (18.4–39.5)	29.9 (19.8–45.0)	18.2 (13.4–24.8)	30.3 (23.1–39.8)	16.1 (11.9–21.9)	45.2 (35.4–57.6)	18.8 (10.4–34.0)	*Increase* (38.2%, *p* = 0.094)	30.1 (Z = −2.08, *p* = 0.037)^*^
T. Taveta	9.4 (2.0–43.3)	0	0	0	0	0	0	0.2 (0–1.7)	100 (Z = −4.53, *p* < 0.001)^*^	*Increase* (20.0%, *p* = 0.168)
Vihiga	40.0 (33.2–48.2)	45.2 (37.6–54.4)	27.7 (20.3–37.7)	34.6 (25.1–47.6)	38.8 (23.5–63.9)	28.2 (17.3–45.8)	29.0 (16.9–49.8)	30.4 (18.7–49.6)	27.4 (Z = −1.12, *p* = 0.263)	32.7 (Z = −2.12, *p* = 0.034)^*^
Wajir	-^ns^	-^ns^	-^ns^	-^ns^	-^ns^	-^ns^	0	0	-^ns^	-^ns^
**Overall**	**22.5 (19.3–26.3)**	**20.6 (18.1–23.4)**	**16.4 (13.5–19.9)**	**13.7 (11.8–15.8)**	**15.3 (12.5–18.8)**	**10.5 (8.8–12.6)**	**13.9 (10.0–19.4)**	**9.4 (7.2–12.2)**	**38.2 (Z** **=** **−2.97**, ***p*** **=** 0.003)^*^	**54.3 (Z** **=** **−7.01**, ***p*** **<** 0.001)^*^

*-^ns^Indicates counties which had not had routine parasitological monitoring, hence, surveys were not conducted in these counties during Year 1, Year 3, and Year 5 assessments*.

* *Indicates statistically significant relative reductions since Year 1 (noting that this could be a reflection of sampling technique, and noting also that oftentimes reductions have not been sustained)*.

*Trichuris trichiura* was the second most prevalent STH species among PSAC in three of four surveys and least prevalent in Y1. *T. trichiura* prevalence was; Y1 5.9% (95% CI: 4.3–8.1); Y3 4.2% (95% CI: 2.6–6.7); Y5 4.5% (95% CI: 3.0–6.8); and Y6 4.8% (95% CI: 2.5–9.0), with a non-significant overall relative reduction (RR = 19.2%, *p* = 0.426). Overall *T. trichiura* prevalence among PSAC appeared to steadily increase after the initial reduction between Y1 and Y3. Counties showed differing levels of infection prevalence. At Y6, three counties showed highest PSAC *T. trichiura* prevalence of over 20%; Busia (28.6%), Homabay (28.6%), and Narok (23.0%). Further, during Y6, nine counties had reduced their prevalence to zero and three counties i.e., Bomet, Migori, and Nyamira, showed a statistically significant relative reduction in prevalence of over 90%. However, infection prevalence increased (significantly) in three counties; Busia, Homabay, and Kilifi (Table 4). Comparison of the county-level trends in PSAC pre-treatment *T. trichiura* prevalence and treatment coverage is outlined in Figure 2C.

**Table 4:** Preschool (PSAC) and school (SAC) aged pre-treatment prevalence % (95%CI) and relative reductions % (Wald test: Z-statistic, *p*-value) for *Trichuris trichiura* infection in 20 counties in Kenya.

**County**	**Year 1**		**Year 3**		**Year 5**		**Year 6**		**Relative Reduction (Year 1–Year 6) RR% (Wald test**, ***p*****-value)**	
	**PSAC**	**SAC**	**PSAC**	**SAC**	**PSAC**	**SAC**	**PSAC**	**SAC**	**PSAC**	**SAC**
Bomet	9.1 (5.2–15.8)	3.7 (1.8–7.2)	7.1 (3.4–14.8)	5.7 (2.7–11.7)	5.0 (1.9–13.1)	4.4 (2.7–7.2)	0	1.2 (0.3–4.7)	100 (Z = −8.49, *p* < 0.001)^*^	66.9 (Z = −1.96, *p* = 0.050)
Bungoma	0	0.9 (0.5–1.7)	0	0	0	0.2 (0.1–0.8)	0	0	0	100 (Z = −14.21, *p* < 0.001)^*^
Busia	7.6 (2.9–19.9)	12.7 (8.2–19.6)	9.9 (4.8–20.6)	14.4 (8.1–25.7)	10.0 (4.7–21.5)	10.2 (5.6–18.5)	28.6 (10.8–75.5)	21.2 (7.3–61.3)	*Increase* (274.6%, *p* = 0.009)^**^	*Increase* (66.8%, *p* = 0.257)
Garissa	-^ns^	-^ns^	-^ns^	-^ns^	-^ns^	-^ns^	0	0	-^ns^	-^ns^
Homa Bay	4.3 (2.4–7.7)	6.0 (4.3–8.4)	0.9 (0.2–3.6)	3.1 (2.1–4.7)	1.7 (0.2–12.3)	3.0 (1.8–4.8)	28.6 (12.3–66.2)	4.6 (2.5–8.2)	*Increase* (569.8%, *p* < 0.001)^**^	24.6 (Z = −0.98, *p* = 0.326)
Kakamega	0.6 (0.1–3.8)	0.7 (0.3–1.7)	0.6 (0.1–3.8)	0.7 (0.3–1.8)	0	0.3 (0.1–0.8)	2.4 (0.2–23.8)	1.2 (0.8–1.8)	*Increase* (331.7%, *p* = 0.310)	*Increase* (78.6%, *p* = 0.233)
Kericho	6.2 (2.6–14.7)	4.5 (2.4–8.6)	7.3 (1.9–28.5)	3.8 (2.1–6.7)	5.5 (1.9–16.0)	4.8 (2.6–9.0)	2.7 (0.1–14.2)	1.2 (0.6–2.3)	56.5 (Z = −0.82, *p* = 0.413)	73.2 (Z = −3.46, *p* = 0.001)^*^
Kilifi	0	1.8 (0.6–5.0)	20.0 (1.9–48.0)	1.8 (0.6–5.1)	0	1.1 (0.2–7.2)	1.8 (0.3–10.7)	4.3 (0.9–20.3)	*Increase* (97.5%, *p* = 0.001)^**^	*Increase* (142.6%, *p* = 0.362)
Kisii	2.0 (0.5–7.6)	1.3 (0.7–2.3)	0	1.2 (0.4–3.3)	1.7 (0.4–7.3)	0.9 (0.5–1.6)	4.3 (1.3–14.2)	1.1 (0.4–2.8)	*Increase* (195.6%, *p* = 0.128)	12.4 (Z = −0.23, *p* = 0.815)
Kisumu	6.6 (2.6–16.8)	3.6 (1.9–6.9)	1.1 (0.2–7.4)	2.2 (1.4–3.4)	2.6 (0.6–12.2)	1.8 (1.1–3.1)	7.1 (1.2–41.4)	1.9 (0.7–5.6)	*Increase* (7.7%, *p* = 0.928)	47.0 (Z = −1.06, *p* = 0.289)
Kitui	-^ns^	-^ns^	-^ns^	-^ns^	-^ns^	-^ns^	0	0	-^ns^	-^ns^
Kwale	6.1 (1.8–21.0)	6.3 (4.0–9.8)	1.9 (0.3–12.1)	3.3 (1.1–10.1)	3.2 (1.3–7.9)	1.6 (0.7–4.0)	12.1 (7.0–21.1)	3.4 (1.2–9.8)	*Increase* (98.0%, *p* = 0.264)	45.8 (Z = −1.14, *p* = 0.252)
Makueni	-^ns^	-^ns^	-^ns^	-^ns^	-^ns^	-^ns^	0	0	-^ns^	-^ns^
Migori	1.6 (0.2–12.5)	0.6 (0.1–2.5)	0	0.1 (0–0.9)	0	0.3 (0–1.9)	0	0.6 (0.2–2.5)	100 (Z = −3.93, *p* < 0.001)^*^	*Increase* (16.3%, *p* = 0.887)
Mombasa	14.6 (3.7–58.6)	17.9 (9.7–33.3)	10.5 (7.7–14.3)	1.8 (0.5–6.9)	0	1.8 (0.2–17.7)	3.0 (0.5–17.6)	0.9 (0.3–2.8)	79.3 (Z = −1.53, *p* = 0.127)	95.1 (Z = −5.08, *p* < 0.001)^*^
Narok	31.3 (19.9–49.2)	29.9 (20.8–43.1)	29.3 (19.3–44.5)	26.5 (17.3–40.6)	26.5 (14.4–48.7)	29.1 (21.2–40.0)	23.0 (12.9–40.7)	13.6 (7.0–26.4)	26.7 (Z = −1.26, *p* = 0.208)	54.6 (Z = −2.73, *p* = 0.006)^*^
Nyamira	3.0 (0.4–21.6)	3.1 (0.6–16.6)	0	0.5 (0.3–1.0)	0	0.4 (0.1–1.9)	0	0.9 (0.2–3.8)	100 (Z = −3.48, *p* = 0.001)^*^	70.8 (Z = −0.99, *p* = 0.323)
T. Taveta	0	2.1 (0.7–5.9)	0	0	2.9 (0.3–33.4)	0	0	0	0	100 (Z = −7.31, *p* < 0.001)^*^
Vihiga	8.2 (2.4–28.7)	10.2 (5.3–19.7)	3.1 (0.5–19.9)	7.6 (4.0–14.5)	7.5 (3.8–14.8)	6.5 (2.6–16.5)	3.2 (0.5–22.9)	8.1 (3.8–17.3)	60.8 (Z = −1.82, *p* = 0.068)	21.2 (Z = −0.87, *p* = 0.386)
Wajir	-^ns^	-^ns^	-^ns^	-^ns^	-^ns^	-^ns^	0	0	-^ns^	-^ns^
**Overall**	**5.9 (4.3–8.1)**	**6.3 (5.0–8.0)**	**4.2 (2.6–6.7)**	**5.1 (3.8–6.9)**	**4.5 (3.0–6.8)**	**4.6 (3.4–6.2)**	**4.8 (2.5–9.0)**	**3.5 (2.2–5.7)**	**19.2 (Z** **=** **−0.80**, ***p*** **=** **0.426)**	**44.3 (Z** **=** **−2.82**, ***p*** **=** 0.005)^*^

*-^ns^Indicates counties which had not had routine parasitological monitoring, hence, surveys were not conducted in these counties during Year 1, Year 3, and Year 5 assessments*.

* *Indicates statistically significant relative reductions since Year 1 (noting that this could be a reflection of sampling technique, and noting also that oftentimes reductions have not been sustained)*.

** *Indicates statistically significant increase in prevalence since Year 1*.

Hookworm was the least prevalent STH detected among PSAC across all years with the exception of Y1 where it was the second most. Prevalence of hookworm among PSAC reduced steadily across all surveys; Y1 13.2% (95% CI: 11.0–15.9); Y3 1.8% (95% CI: 1.1–2.8); Y5 1.2% (95% CI: 0.8–1.9); and Y6 0.5% (95% CI: 0.2–1.3), with an overall significant relative reduction (RR = 96.1%, *p* < 0.001). Although, not representative at the county level, estimates suggested 16 counties had reduced hookworm prevalence to zero during the Y6 survey, and the remaining four counties had prevalence between 1.4 and 2.4%. This resulted in 13 counties significantly reducing the infection prevalence by over 90% (Table 5). Comparison of the county-level trends in PSAC pre-treatment hookworm prevalence and treatment coverage is outlined in Figure 2D.

**Table 5:** Preschool (PSAC) and school (SAC) aged pre-treatment prevalence % (95%CI) and relative reductions % (Wald test: Z-statistic, *p*-value) for hookworm infection in 20 counties in Kenya.

**County**	**Year 1**		**Year 3**		**Year 5**		**Year 6**		**Relative Reduction (Year 1 – Year 6) RR% (Wald test**, ***p*****-value)**	
	**PSAC**	**SAC**	**PSAC**	**SAC**	**PSAC**	**SAC**	**PSAC**	**SAC**	**PSAC**	**SAC**
Bomet	0	0.2 (0–0.6)	0	0.1 (0–0.6)	0	0.1 (0–0.6)	0	0	0	100 (Z = −9.42, *p* < 0.001)^*^
Bungoma	45.2 (34.8–58.5)	44.0 (37.0–52.3)	1.0 (0.1–8.0)	1.7 (0.7–4.4)	0.8 (0.1–5.0)	0.8 (0.4–1.7)	1.7 (0.3–11.3)	0	96.2 (Z = −3.52, *p* < 0.001)^*^	100 (Z = −9.29, *p* < 0.001)^*^
Busia	16.1 (10.5–24.6)	21.0 (16.7–26.4)	4.5 (2.1–9.9)	3.0 (1.8–5.0)	1.2 (0.4–3.4)	3.0 (2.0–4.4)	0	1.2 (0.2–8.6)	100 (Z = −8.43, *p* < 0.001)^*^	94.2 (Z = −3.05, *p* = 0.002)^*^
Garissa	-^ns^	-^ns^	-^ns^	-^ns^	-^ns^	-^ns^	0	0	-^ns^	-^ns^
Homa Bay	8.5 (5.1–14.2)	15.8 (12.9–19.3)	6.2 (3.6–10.6)	5.1 (3.4–7.7)	1.7 (0.5–5.5)	2.7 (1.4–5.2)	0	5.7 (2.8–11.7)	100 (Z = −9.45, *p* < 0.001)^*^	63.8 (Z = −2.90, *p* = 0.004)^*^
Kakamega	27.1 (20.0–36.8)	22.2 (16.7–29.4)	0	0.9 (0.4–1.7)	2.0 (0.7–6.2)	0.3 (0.1–0.8)	2.4 (0.3–19.2)	2.7 (2.1–3.4)	91.0 (Z = −2.49, *p* = 0.013)^*^	88.0 (Z = −12.12, *p* < 0.001)^*^
Kericho	5.6 (2.2–14.0)	5.9 (3.1–11.1)	1.0 (0.2–6.5)	0	0	0.2 (0–0.7)	0	0.4 (0.1–3.0)	100 (Z = −6.15, *p* < 0.001)^*^	93.1 (Z = −3.08, *p* = 0.002)^*^
Kilifi	28.0 (18.6–42.1)	31.2 (29.3–33.1)	0	3.2 (2.2–4.7)	0	1.1 (0.9–1.3)	0	0.7 (0.2–2.8)	100 (Z = −6.12, *p* < 0.001)^*^	97.8 (Z = −5.52, *p* < 0.001)^*^
Kisii	9.9 (4.5–21.9)	11.0 (7.0–17.3)	0	1.5 (0.9–2.6)	0.9 (0.1–6.1)	0.9 (0.5–1.4)	1.4 (0.2–9.2)	0.2 (0–1.7)	85.4 (Z = −2.26, *p* = 0.024)^*^	98.0 (Z = −3.83, *p* < 0.001)^*^
Kisumu	11.0 (5.3–23.2)	7.9 (5.0–12.5)	1.1 (0.2–7.2)	0.3 (0.1–1.3)	0	0.7 (0.3–1.8)	0	0.2 (0–1.4)	100 (Z = −5.81, *p* < 0.001)^*^	97.6 (Z = −4.11, *p* < 0.001)^*^
Kitui	-^ns^	-^ns^	-^ns^	-^ns^	-^ns^	-^ns^	0	0.4 (0.1–1.3)	-^ns^	-^ns^
Kwale	20.4 (11.6–36.0)	25.8 (18.8–35.4)	5.8 (2.9–11.4)	13.8 (8.7–22.1)	3.7 (2.5–5.6)	3.0 (2.0–4.6)	0	3.0 (1.4–6.4)	100 (Z = −5.49, *p* < 0.001)^*^	88.5 (Z = −5.95, *p* < 0.001)^*^
Makueni	-^ns^	-^ns^	-^ns^	-^ns^	-^ns^	-^ns^	0	0.6 (0.2–2.1)	-^ns^	-^ns^
Migori	16.5 (10.1–27.2)	20.8 (16.5–26.0)	0	0.8 (0.4–1.5)	0	0.4 (0.2–1.1)	1.5 (0.2–10.7)	0.6 (0.2–2.3)	90.8 (Z = −2.39, *p* = 0.017)^*^	96.9 (Z = −5.23, *p* < 0.001)^*^
Mombasa	4.9 (0.5–52.0)	7.9 (1.8–35.3)	0	0.7 (0.3–1.7)	0	0	0	0.9 (0.1–6.0)	100 (Z = −2.50, *p* = 0.012)^*^	88.9 (Z = −1.95, *p* = 0.052)
Narok	4.0 (1.6–10.0)	5.3 (2.3–11.8)	0	0.9 (0.5–1.7)	1.2 (0.1–10.3)	1.3 (0.6–2.7)	0	0	100 (Z = −6.96, *p* < 0.001)^*^	100 (Z = −7.15, *p* < 0.001)^*^
Nyamira	5.0 (1.3–18.7)	1.6 (0.6–4.1)	0	0.4 (0.2–0.9)	0	0.1 (0–0.7)	0	0.5 (0.1–3.3)	100 (Z = −4.44, *p* < 0.001)^*^	70.8 (Z = −1.22, *p* = 0.224)
T. Taveta	0	0	0	0	0	0	0	0	0	0
Vihiga	17.6 (10.2–30.6)	15.7 (8.8–27.9)	0	2.0 (1.1–3.8)	2.7 (1.0–7.6)	2.1 (0.9–5.0)	0	1.0 (0.5–1.9)	100 (Z = −6.17, *p* < 0.001)^*^	93.6 (Z = −4.91, *p* < 0.001)^*^
Wajir	-^ns^	-^ns^	-^ns^	-^ns^	-^ns^	-^ns^	0	0	-^ns^	-^ns^
**Overall**	**13.2 (11.0–15.9)**	**15.3 (13.3–17.5)**	**1.8 (1.1–2.8)**	**2.4 (1.8–3.2)**	**1.2 (0.8–1.9)**	**1.3 (1.0–1.7)**	**0.5 (0.2–1.3)**	**1.0 (0.7–1.6)**	**96.1 (Z** **=** **−6.73**, ***p*** **<** 0.001)^*^	**93.3 (Z** **=** **−12.34**, ***p*** **<** 0.001)^*^

*-^ns^Indicates counties which had not had routine parasitological monitoring, hence, surveys were not conducted in these counties during Year 1, Year 3, and Year 5 assessments*.

* *Indicates statistically significant relative reductions since Year 1 (noting that this could be a reflection of sampling technique, and noting also that oftentimes reductions have not been sustained)*.

### SAC Infection Prevalence

Overall, 33.6% (95% CI: 31.2–36.1%), 18.4% (95% CI: 16.2–20.9%), 14.7% (95% CI: 12.6–17.1%) and 12.5% (95% CI: 10.0–15.6%) of SAC children were infected with at least one STH species during Y1, Y3, Y5, and Y6 surveys, respectively, with an overall significant relative reduction of 62.6% (*p* < 0.001) (Table 1). Similarly SAC prevalence of undifferentiated STH differed within counties with highest prevalence, during Y6, observed in Vihiga (30.8%) followed by Bomet (24.2%), Kakamega (23.8%), Busia (23.4%), and Narok (23.1%) and no infections detected in Garissa and Wajir counties. However, Kitui, Makueni, and Taita had their prevalence below 1%. County level relative reduction indicated that only two counties; Bungoma and Migori, reduced the SAC prevalence by over 90% (Table 1). Comparison of the county-level trends in SAC pre-treatment undifferentiated STH prevalence and treatment coverage is outlined in Figure 2A.

*Ascaris lumbricoides* remained the most prevalent STH species among SAC during all the four survey time points despite the prevalence steadily declining. Prevalence for *A. lumbricoides* was estimated at; Y1 20.6% (95% CI: 18.1–23.4); Y3 13.7% (95% CI: 11.8–15.8); Y5 10.5% (95% CI: 8.8–12.6); and Y6 9.4% (95% CI: 7.2–12.2), with a statistically significant overall relative reduction (RR = 54.3%, *p* < 0.001). Further, we noted that the overall prevalence of *A. lumbricoides* among SAC was lower than PSAC prevalence at each respective survey time point. Similarly, counties showed differing level of infection prevalence. At Y6, the counties with higher SAC *A. lumbricoides* prevalence (>20%) were only Vihiga (30.4%) and Bomet (23.6%). This demonstrated fewer counties with SAC prevalence >20% compared to PSAC, who recorded seven counties with prevalence >20%. Only one county, Kwale, statistically significantly *reduced A. lumbricoides* prevalence by over 90% (Table 3). Comparisons of county-level trends in SAC pre-treatment *A. lumbricoides* prevalence and treatment coverage is outlined in Figure 2B.

*Trichuris trichiura* was the second most prevalent STH species among SAC for three surveys but least prevalent during Y1. Prevalence of *T. trichiura* for SAC was estimated at; Y1 6.3% (95% CI: 5.0–8.0); Y3 5.1% (95% CI: 3.8–6.9); Y5 4.6% (95% CI: 3.4–6.2); and Y6 3.5% (95% CI: 2.2–5.7), with a statistically significant overall relative reduction (RR = 44.3%, *p* = 0.005). The reduction in the *T. trichiura* prevalence appeared steady from Y1 to Y6. Counties showed differing levels of prevalence (Table 4). Comparison of the county-level trends in SAC pre-treatment *T. trichiura* prevalence and treatment coverage is outlined in Figure 2C.

Hookworm was the least prevalent form of STH infection among SAC in all the surveys except Y1, where it was second. Prevalence of hookworm among SAC reduced steadily across all surveys; Y1 15.3% (95% CI: 13.3–17.5); Y3 2.4% (95% CI: 1.8–3.2); Y5 1.3% (95% CI: 1.0–1.7); and Y6 1.0% (95% CI: 0.7–1.6), with an overall statistically significant relative reduction (RR = 93.3%, *p* < 0.001). Overall SAC hookworm prevalence was higher among SAC than PSAC across all surveys. Similarly, counties showed differing levels of infection prevalence. Six out of the 20 counties reduced hookworm prevalence to zero during the Y6 survey, and remaining counties showed prevalence between 0.2 and 5.7%. Ten counties significantly reduced prevalence by over 90% (Table 5). Comparison of the county-level trends in SAC pre-treatment hookworm prevalence and treatment coverage is outlined in Figure 2D.

### Correlations Between Prevalence and Treatment Coverage for PSAC Group

Data from all surveys suggested significant but a weak negative correlation between treatment coverage and undifferentiated STH prevalence (*r* = −0.144, *p* = 0.002) (Table 6). At Years 3 and 5, no overall significant correlation was observed while at Y6, undifferentiated STH was significantly but weakly positively correlated with treatment coverage (*r* = 0.277, *p* < 0.001). Differing county-level correlations for undifferentiated STH were observed over all time points and during Y3, Y5, and Y6, as shown in Table 6. In one county, Migori, there was a strong (*r* > 0.7) significant negative correlation between prevalence and treatment coverage overall; nine other counties had a weak (*r* < 0.7) significant negative correlation overall. Ten of the 20 counties showed no overall significant correlation. At Y3, no strong significant negative or positive correlation was seen in any county, but weak negative and positive correlations were seen in four and three counties, respectively. At Y5, no strong correlations were seen as well, but weak negative correlations were seen in three counties. At Y6, a strong negative correlation was seen in Kisii (*r* = −0.712, *p* < 0.001), with weak negative correlation seen in Bungoma County and strong positive correlation seen in Bomet County. Whilst the general trend amongst those correlations that were significant was for negative rather than positive correlations, only two counties, Narok and Vihiga, showed statistically significant negative correlations across each consecutive time point (Y3 and Y6). Further, frequently there were insufficient PSAC observations to analyze correlations at county level at separate time points.

**Table 6:** Overall and survey time point correlations between pre-treatment undifferentiated STH prevalence of infection and previous year treatment coverage by county among pre-school (PSAC) and school (SAC) aged group of children in Kenya.

**County/survey^$^**	**Overall (All time points)**		**Year 3**		**Year 5**		**Year 6**	
	**Correlation(*****r*****)**, ***p*****-value**		**Correlation(*****r*****)**, ***p*****-value**		**Correlation(*****r*****)**, ***p*****-value**		**Correlation(*****r*****)**, ***p*****-value**	
	**PSAC**	**SAC**	**PSAC**	**SAC**	**PSAC**	**SAC**	**PSAC**	**SAC**
Bomet	*r = –*0.266, *p* = 0.043^*^	*r = –*0.341, *p* < 0.001^*^	*r = –*0.482, *p* = 0.037^*^	***r=–*****0.718**, ***p*** **<** 0.001^*^^°^	*r* = −0.186, *p* = 0.491	*r = –*0.698, *p* < 0.001^*^	*r =* 0.503, *p* = 0.045^#^	*r =* 0.229, *p* = 0.456
Bungoma	*r = –*0.354, *p* = 0.013^*^	*r = –*0.525, *p* < 0.001^*^	*r = –*0.171, *p* = 0.594	*r = –*0.374, *p* = 0.226	*r =* 0.081, *p* = 0.808	*r = –*0.134, *p* = 0.693	*r = –*0.566, *p* = 0.015^*^	*r =* 0.187, *p* = 0.734
Busia	*r = –*0.075, *p* = 0.580	*r = –*0.189, *p* = 0.127	*r =* 0.255, *p* = 0.343	*r = –*0.038, *p* = 0.861	*r = –*0.199, *p* = 0.095	*r = –*0.522, *p* < 0.001^*^	Insufficient obs	Insufficient obs
Garissa	-^ns^	-^ns^	-^ns^	-^ns^	-^ns^	-^ns^	Insufficient obs	Insufficient obs
Homa Bay	*r = –*0.034, *p* = 0.793	*r = –*0.169, *p* = 0.279	*r =* 0.272, *p* = 0.023^#^	*r =* 0.232, *p* = 0.251	*r = –*0.098, *p* = 0.674	*r = –*0.077, *p* = 0.718	Insufficient obs	***r=–*****0.831**, ***p*** **<** 0.001^*^^°^
Kakamega	*r = –*0.366, *p* = 0.004^*^	*r = –*0.383, *p* < 0.001^*^	*r =* 0.516, *p* < 0.001^#^	*r =* 0.014, *p* = 0.956	*r = –*0.394, *p* = 0.022^*^	*r = –*0.552, *p* < 0.001^*^	*r =* 0.196, *p* = 0.600	*r = –*0.429, *p* = 0.201
Kericho	*r = –*0.205, *p* = 0.341	*r = –*0.159, *p* = 0.477	*r =* 0.029, *p* = 0.933	*r = –*0.253, *p* = 0.287	*r = –*0.033, *p* = 0.910	*r = –*0.167, *p* = 0.528	*r =* 0.450, *p* = 0.028	*r = –*0.637, *p* = 0.011^*^
Kilifi	*r = –*0.543, *p* = 0.024^*^	*r =* 0.357, *p* < 0.001^#^	Insufficient obs	Insufficient obs	Insufficient obs	Insufficient obs	Insufficient obs	*r = –*0.209, *p* = 0.329
Kisii	*r = –*0.186, *p* = 0.180	*r = –*0.467, *p* < 0.001^*^	*r =* 0.152, *p* = 0.664	*r = –*0.253, *p* = 0.150	*r = –*0.188, *p* = 0.540	*r = –*0.147, *p* = 0.636	***r=–*****0.712**, ***p*** **<** 0.001^*^^°^	*r =* 0.415, *p* = 0.108
Kisumu	*r = –*0.507, *p* < 0.001^*^	*r =* 0.162, *p* = 0.082	*r =* 0.327, *p* = 0.028^#^	***r=*** **0.847**, ***p*** **<** **0.001^#^^°^**	*r = –*0.471, *p* = 0.060	*r = –*0.642, *p* < 0.011^*^	*r =* 0.086, *p* = 0.792	*r =* 0.100, *p* = 0.443
Kitui	-^ns^	-^ns^	-^ns^	-^ns^	-^ns^	-^ns^	Insufficient obs	Insufficient obs
Kwale	*r = –*0.266, *p* = 0.018^*^	*r =* 0.312, *p* < 0.001^#^	*r =* 0.095, *p* = 0.689	***r=–*****0.776**, ***p*** **<** 0.001^*^^°^	*r =* 0.111, *p* = 0.640	*r =* 0.695, *p* < 0.001^*^	Insufficient obs	Insufficient obs
Makueni	-^ns^	-^ns^	-^ns^	-^ns^	-^ns^	-^ns^	Insufficient obs	Insufficient obs
Migori	***r=–*****0.712**, ***p*** **<** 0.001^*^^°^	*r =* 0.584, *p* = 0.001^#^	Insufficient obs	Insufficient obs	Insufficient obs	Insufficient obs	Insufficient obs	Insufficient obs
Mombasa	*r = –*0.481, *p* < 0.001^*^	*r =* 0.114, *p* = 0.421	Insufficient obs	*r = –*0.461, *p* = 0.242	Insufficient obs	*r =* 0.500, *p* = 0.182	*r = –*0.250, *p* = 0.191	*r = –*0.250, *p* = 0.342
Narok	*r = –*0.443, *p* = 0.003^*^	*r =* 0.218, *p* = 0.183	*r = –*0.449, *p* = 0.043^*^	***r=–*****0.759**, ***p*** **<** 0.001^*^^°^	*r = –*0.552, *p* = 0.020^*^	*r = –*0.420, *p* = 0.081	Insufficient obs	Insufficient obs
Nyamira	*r =* 0.011, *p* = 0.950	*r =* 0.164, *p* = 0.140	*r = –*0.326, *p* = 0.047^*^	*r =* 0.225, *p* = 0.111	*r = –*0.342, *p* = 0.034	*r =* 0.060, *p* = 0.599	*r = –*0.222, *p* = 0.552	*r =* 0.345, *p* = 0.216
Taita Taveta	*r = –*0.185, *p* = 0.684	*r = –*0.670, *p* < 0.001^*^	Insufficient obs	Insufficient obs	Insufficient obs	Insufficient obs	Insufficient obs	***r=–*****0.980**, ***p*** **<** 0.001^*^^°^
Vihiga	*r = –*0.462, *p* < 0.001^*^	*r = –*0.487, *p* = 0.025^*^	*r = –*0.614, *p* = 0.002^*^	*r = –*0.633, *p* = 0.016^*^	*r = –*0.681, *p* < 0.001^*^	*r = –*0.632, *p* = 0.007^*^	*r = –*0.236, *p* = 0.669	*r = –*0.286, *p* = 0.515
Wajir	-^ns^	-^ns^	-^ns^	-^ns^	-^ns^	-^ns^	Insufficient obs	Insufficient obs
**Overall**	***r=–*****0.144**, ***p*** **=** 0.002^*^	***r=*** **0.053**, ***p*** **=** **0.409**	***r=*** **0.114**, ***p*** **=** **0.096**	***r=–*****0.136**, ***p*** **=** **0.121**	***r=–*****0.105**, ***p*** **=** **0.241**	***r=–*****0.160**, ***p*** **=** **0.088**	***r=*** **0.277**, ***p*** **<** **0.001^#^**	***r=*** **0.330**, ***p*** **<** **0.001^#^**

$ *Year 1 correlations were not included since there was no previous treatment coverage to compare with the Year 1 prevalence*.

*-^ns^Indicates counties which had not had routine parasitological monitoring, hence, surveys were not conducted in these counties during Year 1, Year 3, and Year 5 assessments*.

* *Indicates a statistically significant negative correlation*.

# *Indicates a statistically significant positive correlation*.

° *Indicates a strong significant correlation (defined as r > |0.7|, p <0.001) (i.e. values in gray shade)*.

For differentiated STH across all time points, treatment coverage was only significantly (but weakly) negatively correlated with hookworm infection (*r* = −0.307, *p* < 0.001) while no significant correlations were found between either *A. lumbricoides* (*r* = −0.081, *p* = 0.111) or *T. trichiura* (*r* = −0.016, *p* = 0.697) and treatment coverage. Across all time points, hookworm was strongly, significantly and negatively correlated with treatment coverage in two counties; Kilifi (*r* = −0.809, *p* < 0.001) and Migori (*r* = −0.716, *p* < 0.001), but weakly, significantly and negatively correlated with treatment coverage in 10 counties. *A. lumbricoides* was weakly, significantly and negatively correlated with treatment coverage in three counties; Narok (*r* = −0.319, *p* = 0.011), Taita Taveta (*r* = −0.501, *p* = 0.039), and Vihiga (*r* = −0.420, *p* < 0.001). Similarly, *T. trichiura* was weakly, significantly and negatively correlated with treatment coverage in two counties; Mombasa (*r* = −0.481, *p* < 0.001) and Vihiga (*r* = −0.286, *p* = 0.003), and weakly, significantly and positively correlated in two counties; Kilifi (*r* = 0.100, *p* = 0.009) and Taita Taveta (*r* = 0.296, *p* = 0.034) (Table 7).

**Table 7:** Overall correlations between pre-treatment differentiated STH prevalence of infection and previous year treatment coverage by county among pre-school (PSAC) and school (SAC) aged group of children in Kenya.

**County/Survey^$^**	**Overall (All time points)**					
	***A. lumbricoides***		**Hookworm**		***T. trichiura***	
	**PSAC**	**SAC**	**PSAC**	**SAC**	**PSAC**	**SAC**
Bomet	*r = –*0.230, *p* = 0.077	*r = –*0.330, *p* = 0.001^*^	Insufficient obs	*r =* 0.057, *p* = 0.247	*r = –*0.119, *p* = 0.523	*r = –*0.241, *p* = 0.187
Bungoma	*r = –*0.312, *p* = 0.053	*r = –*0.587, *p* = 0.001^*^	*r = –*0.347, *p* = 0.002^*^	*r = –*0.501, *p* = 0.001^*^	Insufficient obs	*r = –*0.303, *p* = 0.133
Busia	*r = –*0.078, *p* = 0.562	*r = –*0.105, *p* = 0.372	*r = –*0.436, *p* < 0.001^*^	*r =* 0.279, *p* = 0.052	*r =* 0.085, *p* = 0.293	*r = –*0.465, *p* < 0.001^*^
Garissa	Insufficient obs	Insufficient obs	Insufficient obs	Insufficient obs	Insufficient obs	Insufficient obs
Homa Bay	*r = –*0.072, *p* = 0.607	*r = –*0.206, *p* = 0.238	*r = –*0.110, *p* = 0.157	*r =* 0.016, *p* = 0.876	*r =* 0.042, *p* = 0.689	*r = –*0.148, *p* = 0.391
Kakamega	*r = –*0.221, *p* = 0.067	*r = –*0.233, *p* = 0.041^*^	*r = –*0.672, *p* < 0.001^*^	*r = –*0.484, *p* < 0.001^*^	*r = –*0.093, *p* = 0.235	*r = –*0.001, *p* = 0.990
Kericho	*r = –*0.165, *p* = 0.451	*r = –*0.189, *p* = 0.394	*r = –*0.181, *p* = 0.029^*^	*r =* 0.101, *p* = 0.382	*r = –*0.186, *p* = 0.191	*r = –*0.157, *p* = 0.506
Kilifi	Insufficient obs	*r = –*0.023, *p* = 0.955	***r=–*****0.809**, *p*<0.001^*^^°^	*r =* 0.451, *p* < 0.001^#^	*r =* 0.100, *p* = 0.009^#^	*r = –*0.184, *p* = 0.069
Kisii	*r =* 0.017, *p* = 0.918	*r = –*0.416, *p* = 0.004^*^	*r = –*0.579, *p* < 0.001^*^	*r = –*0.308, *p* < 0.001^*^	*r = –*0.045, *p* = 0.638	*r = –*0.132, *p* = 0.343
Kisumu	*r = –*0.278, *p* = 0.283	*r =* 0.095, *p* = 0.290	*r = –*0.491, *p* < 0.001^*^	*r =* 0.211, *p* < 0.001^#^	*r = –*0.048, *p* = 0.821	*r =* 0.046, *p* = 0.740
Kitui	Insufficient obs	Insufficient obs	Insufficient obs	Insufficient obs	Insufficient obs	Insufficient obs
Kwale	*r = –*0.082, *p* = 0.131	*r =* 0.184, *p* = 0.008^#^	*r = –*0.208, *p* = 0.037^*^	*r = –*0.358, *p* < 0.001^*^	*r = –*0.177, *p* = 0.169	*r = –*0.010, *p* = 0.943
Makueni	Insufficient obs	Insufficient obs	Insufficient obs	Insufficient obs	Insufficient obs	Insufficient obs
Migori	*r =* 0.112, *p* = 0.555	*r =* 0.280, *p* = 0.009^#^	***r=–*****0.716**, ***p*** **<** 0.001^*^^°^	*r =* 0.590, *p* < 0.001^#^	*r = –*0.289, *p* = 0.053	*r =* 0.222, *p* = 0.057
Mombasa	Insufficient obs	*r =* 0.092, *p* = 0.313	*r = –*0.384, *p* < 0.001^*^	*r = –*0.033, *p* = 0.862	*r = –*0.481, *p* < 0.001^*^	*r = –*0.070, *p* = 0.763
Narok	*r = –*0.319, *p* = 0.011^*^	*r =* 0.274, *p* = 0.037^#^	*r = –*0.037, *p* = 0.494	*r =* 0.237, *p* = 0.093	*r = –*0.230, *p* = 0.116	*r = –*0.090, *p* = 0.671
Nyamira	*r = –*0.089, *p* = 0.632	*r =* 0.177, *p* = 0.125	*r = –*0.341, *p* = 0.006^*^	*r = –*0.014, *p* = 0.826	*r = –*0.270, *p* = 0.055	*r =* 0.013, *p* = 0.694
Taita Taveta	*r = –*0.501, *p* = 0.039^*^	*r =* 0.137, *p* = 0.091	Insufficient obs	Insufficient obs	*r =* 0.296, *p* = 0.034^#^	***r=–*****0.711**, *p*<0.001^*^^°^
Vihiga	*r = –*0.420, *p* < 0.001^*^	*r = –*0.507, *p* = 0.021^*^	*r = –*0.243, *p* = 0.005^*^	*r =* 0.152, *p* = 0.038^#^	*r = –*0.286, *p* = 0.003^*^	*r = –*0.459, *p* = 0.007^*^
Wajir	Insufficient obs	Insufficient obs	Insufficient obs	Insufficient obs	Insufficient obs	Insufficient obs
**Overall**	***r=–*****0.081**, ***p*** **=** **0.111**	***r=–*****0.119**, ***p*** **=** **0.101**	***r=–*****0.307**, ***p*** **<** 0.001^*^	***r=–*****0.044**, ***p*** **=** **0.320**	***r=–*****0.016**, ***p*** **=** **0.697**	***r=*** **0.030**, ***p*** **=** **0.506**

$ *Year 1 correlations were not included since there was no previous treatment coverage to compare with the Year 1 prevalence*.

* *Indicates a statistically significant negative correlation*.

# *Indicates a statistically significant positive correlation*.

° *Indicates a strong significant correlation (defined as r > |0.7|, p <0.001) (i.e. values in gray shade)*.

County-level correlations, for each specific survey time point, for the differentiated STH infections are outlined in the Supplementary Table 1. Over all counties, *A. lumbricoides* prevalence had a significant weak positive correlation with treatment coverage at the Y6 survey (*r* = 0.325, *p* < 0.001) (Supplementary Table 1); there were no other significant correlations (either negative or positive) over all counties seen for any species at any other survey time points. By county, the only strong correlation seen was in Y6 in Kisii, where a significant positive correlation was seen (*r* = 0.824, *p* = 0.001). Whilst other significant negative or positive, correlations were seen within counties at different time points, they were weak, with again no trend emerging across time points for any differentiated STH within any county. Often, there were again insufficient observations to assess this at county level by time point.

*Ascaris lumbricoides* infection was weakly, significantly and negatively correlated with treatment coverage in Bomet (*r* = −0.664, *p* < 0.001), Nyamira (*r* = −0.326, p = 0.047), and Vihiga (*r* = −0.614, *p* = 0.002) counties during Y3; Kakamega (*r* = −0.376, *p* = 0.030), Kisii (*r* = −0.545, *p* = 0.028), Nyamira (*r* = −0.342, *p* = 0.034), and Vihiga (*r* = −0.669, *p* < 0.001) during Y5; and Bungoma (*r* = −0.604, *p* = 0.014) and Kisii (*r* = −0.625, *p* = 0.002) during Y6. However, it was significantly positively correlated with the treatment coverage in Busia (*r* = 0.471, *p* = 0.010) and Kakamega (*r* = 0.516, *p* < 0.001) counties during Y3, and Bomet (*r* = 0.503, *p* = 0.045) and Kericho (*r* = 0.450, *p* = 0.028) during Y6 (Supplementary Table 1).

Hookworm infection showed weak significant negative correlations only in Busia County (*r* = −0.326, *p* = 0.048) during Y3; in Homabay (*r* = −0.312, *p* = 0.006), and Kakamega (*r* = −0.224, *p* = 0.049) during Y5 survey. However, no significant (negative or positive) correlation was observed during Y6 survey (Supplementary Table 1).

*Trichuris trichiura* showed weak significant negative correlations in Kericho (*r* = −0.400, *p* = 0.016) and Narok (*r* = −0.459, *p* = 0.041) counties during Y3 survey; in Kisii (*r* = −0.504, *p* = 0.014) and Narok (*r* = −0.597, *p* = 0.001) during Y5 survey; and a strong negative correlation in Kisii County (*r* = −0.824, *p* < 0.001) during Y6 survey. However, it showed a significant positive correlation in Homabay (*r* = 0.200, *p* = 0.020) during Y3 (Supplementary Table 1).

**Figure 3:**
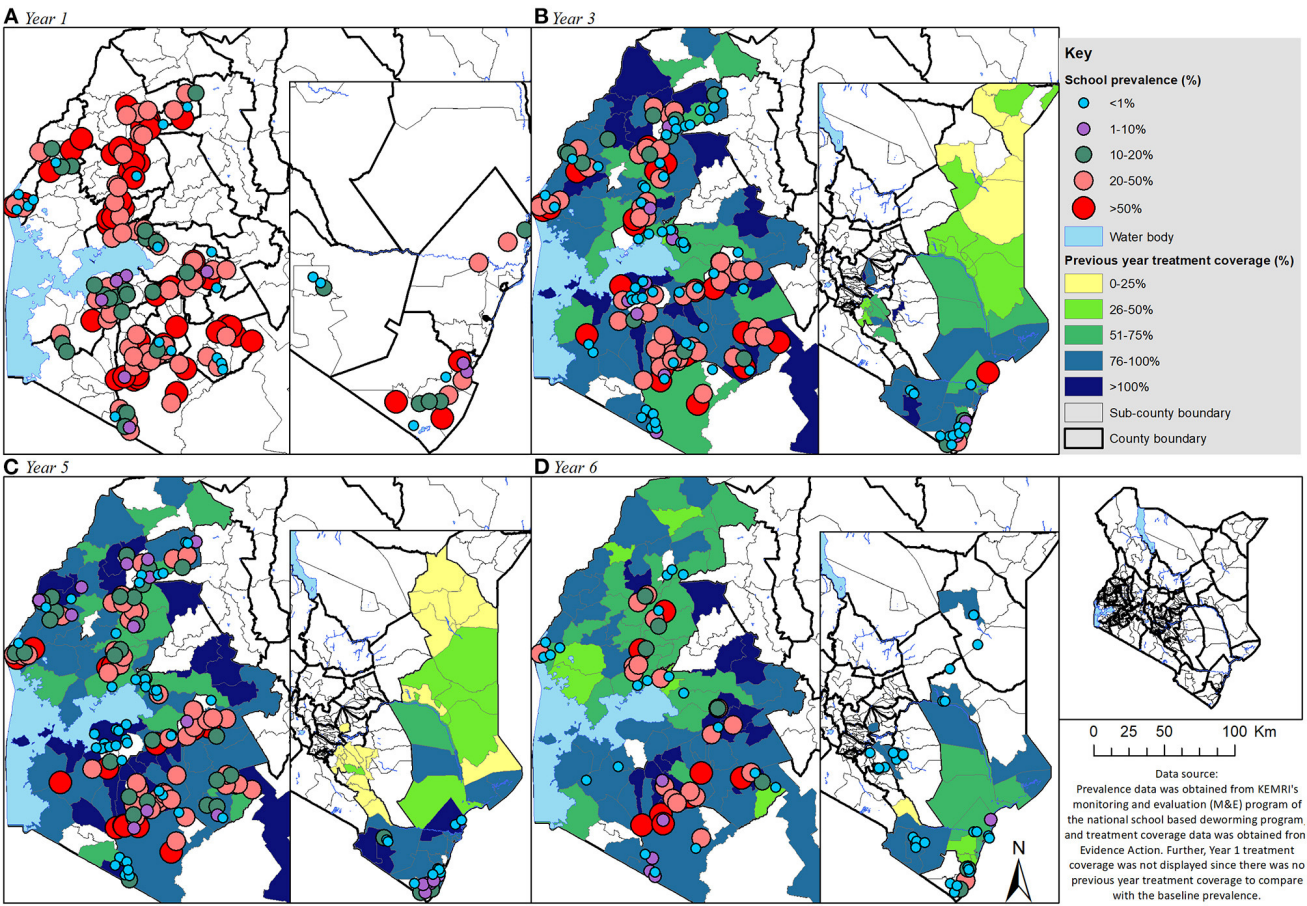
Geographical distribution of the undifferentiated STH pre-treatment prevalence overlaid with the previous year treatment coverage (**A**: Year 1, **B**: Year 3, **C**: Year 5, and **D**: Year 6) for the preschool aged (PSAC) group of children in Kenya.

Geographical distribution of undifferentiated STH pre-treatment prevalence overlaid with the previous year's treatment coverage for the PSAC group is shown in Figure 3, the figure helped to visualize the PSAC county and/or subcounty level correlations between prevalence and treatment coverage. Even though, it can be observed that the PSAC prevalence appeared to have reduced over the years, it was still high especially in many areas in Western parts of Kenya. Further, we noted that the treatment coverage during all the survey years was high (>75%), however, this high coverage did not clearly translate to low PSAC prevalence in the respective areas.

### Correlations Between Prevalence and Treatment Coverage for SAC Group

Table 6 outlines the correlations between pre-treatment undifferentiated STH prevalence and previous year's treatment coverage among SAC for Y3, Y5, and Y6 surveys. A weak significant positive correlation was observed during Y6 (*r* = 0.330, *p* < 0.001), this indicated that prevalence of STH continued to increase even as coverage increased. Nonetheless, these overall correlations mask underlying negative and positive county-level correlations. In overall, weak negative county-level correlations were observed in Bomet (*r* = −0.482, *p* = 0.037), Bungoma (*r* = −0.525, *p* < 0.001), Kakamega (*r* = −0.383, *p* < 0.001), Kisii (*r* = −0.467, *p* < 0.001), Taita Taveta (*r* = −0.670, *p* < 0.001), and Vihiga (*r* = −0.487, *p* = 0.025), with a weak positive correlation in three counties; Kilifi, Kwale, and Migori. During Y3 survey, strong negative correlations were observed in Bomet (*r* = −0.718, *p* < 0.001), Kwale (*r* = −0.776, *p* < 0.001), and Narok (*r* = −0.759, *p* < 0.001), and weak negative correlation in Vihiga, and further, a strong positive correlation observed in Kisumu (*r* = 0.847, *p* < 0.001). During Y5 survey, no strong correlation was observed but weak negative correlations were observed in six counties. During Y6 survey, strong negative correlations were observed in two counties; Homabay (*r* = −0.831, *p* < 0.001) and Taita Taveta (*r* = −0.980, *p* < 0.001), with a weak negative correlation in Kericho.

Overall correlations between *Ascaris lumbricoides* infection prevalence and treatment coverage is shown in Table 7, and this overall correlation was non-significant. However, five counties; Bomet, Bungoma, Kakamega, Kisii, and Vihiga showed weak significant negative correlations, while Kwale and Migori indicated a weak significant positive correlation (Table 7). Specific survey time point correlations are shown in the Supplementary Table 1. During Y3 survey, strong significant negative correlations were observed in Bomet (*r* = −0.727, *p* < 0.001) and Kisumu (*r* = −0.721, *p* = 0.047), a weak significant negative correlation in Vihiga (*r* = −0.613, *p* = 0.029). During Y5, weak significant negative correlations were observed in Bomet (*r* = −0.670, *p* < 0.001), Kakamega (*r* = −0.527, *p* < 0.001), Kisumu (*r* = −0.608, p = 0.013), and Vihiga (*r* = −0.645, p = 0.004). During Y6, a strong significant negative correlation was observed in Taita Taveta (*r* = −0.980, *p* < 0.001), and weak significant negative correlation in Kericho (*r* = −0.675, *p* = 0.002) (Supplementary Table 1).

Overall correlation between hookworm infection prevalence and treatment coverage is shown in Table 7, and this overall correlation was non-significant. However, five counties; Bomet, Bungoma, Kakamega, Kisii, and Vihiga showed overall weak significant negative correlations, while Kilifi, Kisumu, Migori, and Vihiga indicated a weak significant positive correlation (Table 7). Specific survey time point correlations are shown in the Supplementary Table 1. During Y3 survey, weak significant negative correlations were observed in Kwale (*r* = −0.626, *p* = 0.003) and Mombasa (*r* = −0.100, *p* = 0.001), and a weak significant positive correlation in Busia (*r* = 0.456, *p* = 0.005), and Kisumu (*r* = 0.676, *p* < 0.001). During Y5, weak significant negative correlations were observed in Kakamega (*r* = −0.431, *p* < 0.001) and Narok (*r* = −0.421, *p* = 0.008), while weak significant positive correlations were observed in Busia (*r* = 0.692, *p* < 0.001), Kericho (*r* = 0.495, *p* = 0.009), Kisii (*r* = 0.556, *p* = 0.014), and Kwale (*r* = −0.497, *p* = 0.010). During Y6, weak significant negative correlations were observed in Kakamega (*r* = −0.461, *p* = 0.048) and Vihiga (*r* = −0.670, *p* < 0.001) (Supplementary Table 1).

Overall correlation between *Trichuris trichiura* infection prevalence and treatment coverage is shown in Table 7, and this overall correlation was non-significant. However, one county; Taita Taveta (*r* = −0.711, *p* < 0.001), showed a strong significant negative correlation, while Busia and Vihiga showed weak significant negative correlations (Table 7). Specific survey time point correlations are shown in Supplementary Table 1. During Y3 survey, strong significant negative correlations were observed in Kisii (*r* = −0.708, *p* < 0.001) and Kwale (*r* = −0.967, *p* < 0.001), and weak significant negative correlations in Narok (*r* = −0.637, *p* < 0.001), and Vihiga (*r* = −0.617, *p* < 0.001). During Y5, a strong significant negative correlation was observed in Narok (*r* = −0.706, *p* < 0.001) and strong significant positive correlations in Kwale (*r* = 0.798, *p* < 0.001) and Nyamira (*r* = 0.946, *p* < 0.001). Further, weak significant negative correlations were observed in Bomet, Busia, Kakamega, and Vihiga. During Y6, a strong significant negative correlation was observed in Homabay (*r* = −0.903, *p* < 0.001), and weak significant negative correlations in Kisii and Mombasa (Supplementary Table 1).

**Figure 4:**
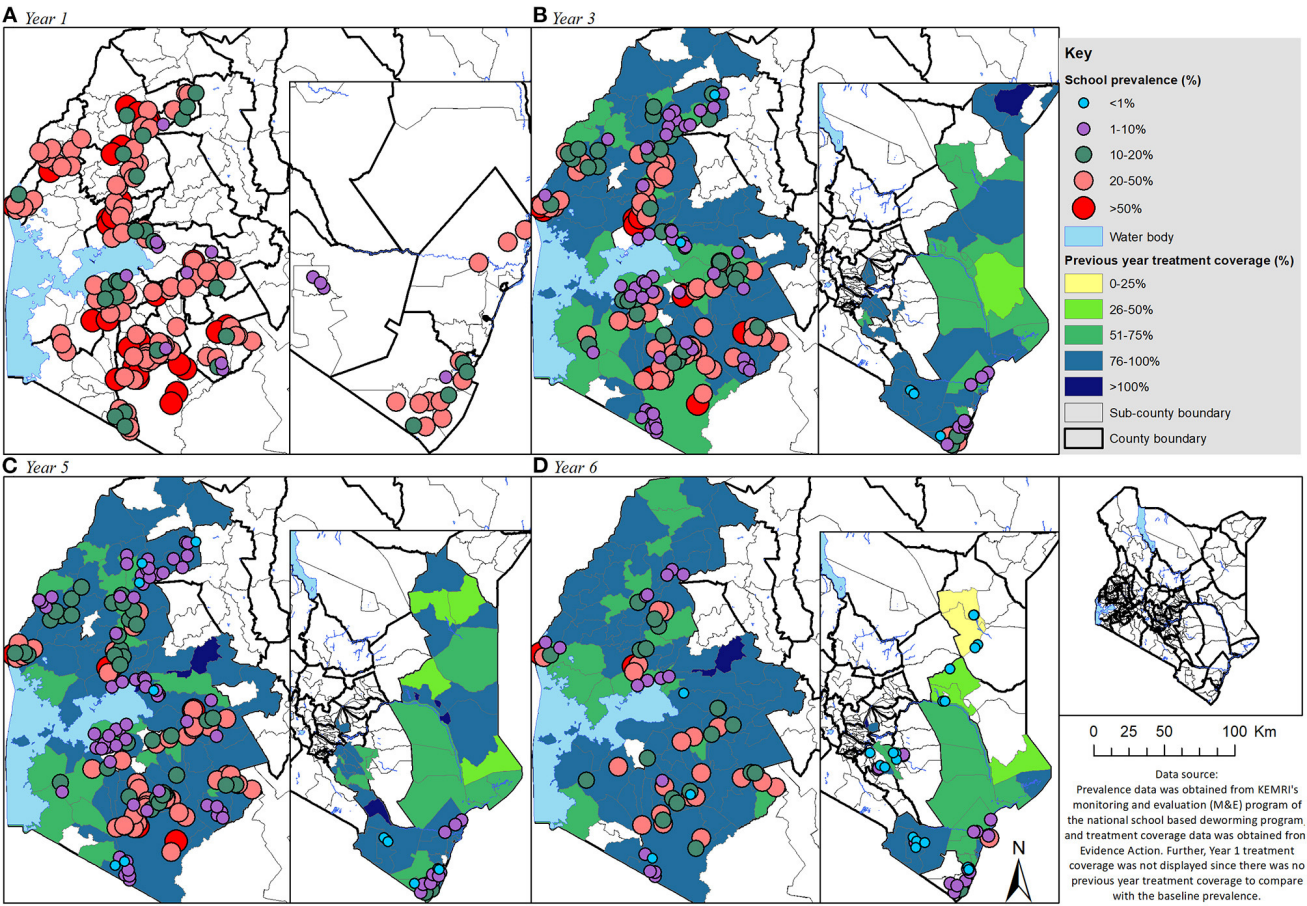
Geographical distribution of the undifferentiated STH pre-treatment prevalence overlaid with the previous year treatment coverage (**A**: Year 1, **B**: Year 3, **C**: Year 5, and **D**: Year 6) for the school aged (SAC) group of children in Kenya.

Geographical distribution of undifferentiated STH pre-treatment prevalence overlaid with the previous year's treatment coverage for the SAC group is shown in Figure 4, the figure helped to visualize the SAC county and/or subcounty level correlations between prevalence and treatment coverage. From the figure, it can be observed that the SAC prevalence had reduced tremendously over the years, in most subcounties, the treatment coverage was high (>75%). Actually, in most areas especially during Y6 where the treatment coverage was above 75%, the prevalence was seen to be below 20% (these areas can be analogously viewed as portraying negative correlations). On the other hand, some areas did show high prevalence of 20% and above despite their subcounty treatment coverage being above 75% (these areas can be seen to portray positive correlations). However, there was no clear pattern of this correlation in the map, indicating heterogeneity in the subcounty/county correlation analysis.

### Minimum Coverage for Sustained Reductions in Prevalence

**Figure 5:**
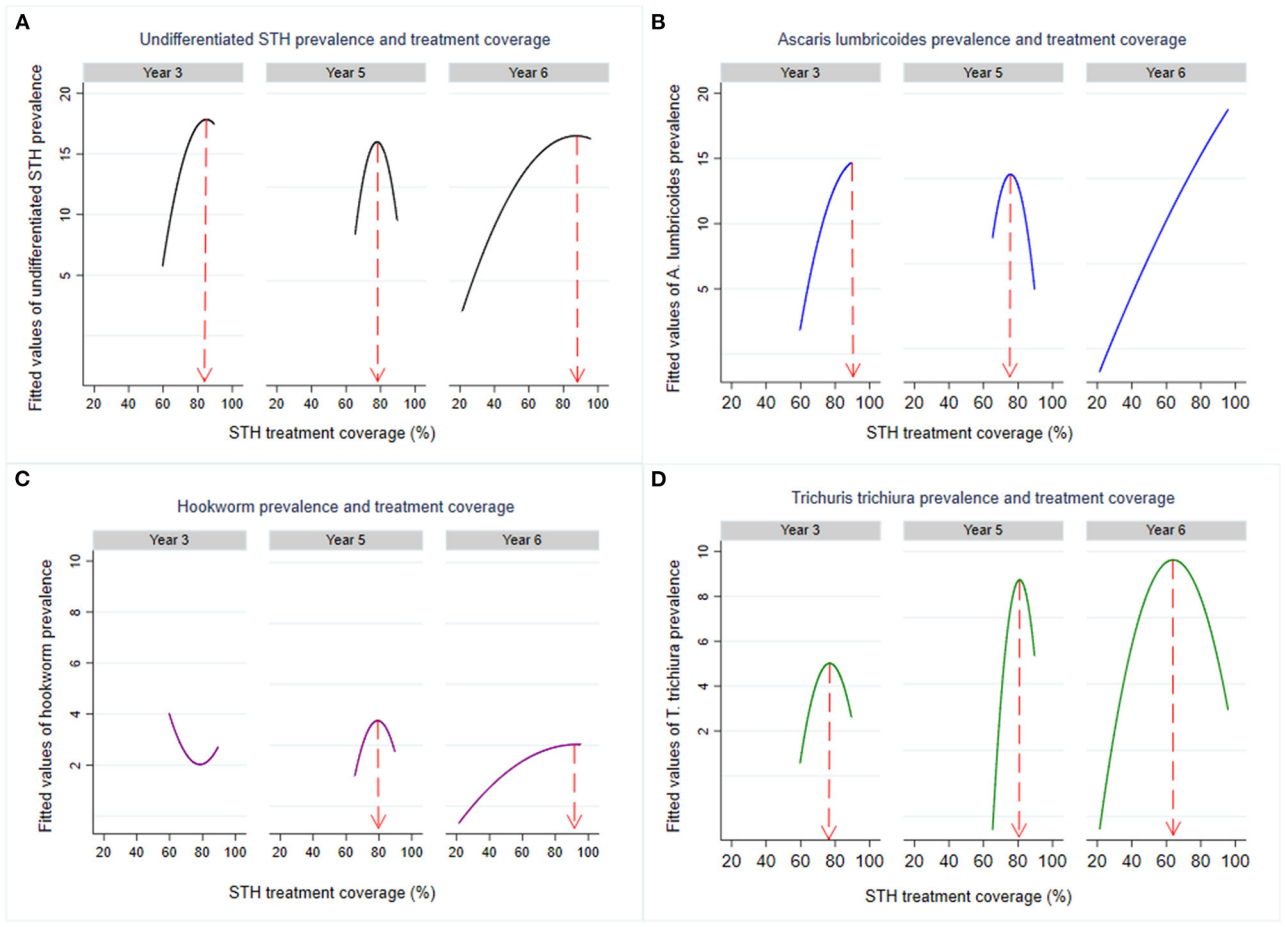
Non-linear relationship between STH infections prevalence (**A**: Undifferentiated STH, **B**: Ascaris lumbricoides, **C**: Hookworm, and **D**: Trichuris trichiura) and treatment coverage for year 1, year 3, and year 6 survey time points among the general children population (both PSAC and SAC combined) in Kenya. Note that Year 1 was not displayed since there was no previous year treatment coverage to compare with the baseline prevalence.

Analysis of the minimum treatment coverage required to ensure a reduction of the infection prevalence over time for both age groups was demonstrated by use of the non-linear relationship curves. Observed non-linear relationships between STH prevalence and previous year treatment coverage is shown in Figure 5. From the figure, it was observed that for program-wide reduction of undifferentiated STH in both PSAC and SAC, a likely minimum treatment coverage of 82% is required. Additionally, for each differentiated STH species, likely minimum treatment coverage of 85% for hookworm, 84% for *A. lumbricoides*, and 74% for *T. trichiura* is required to be maintained.

### Change Point Analysis

**Figure 6:**
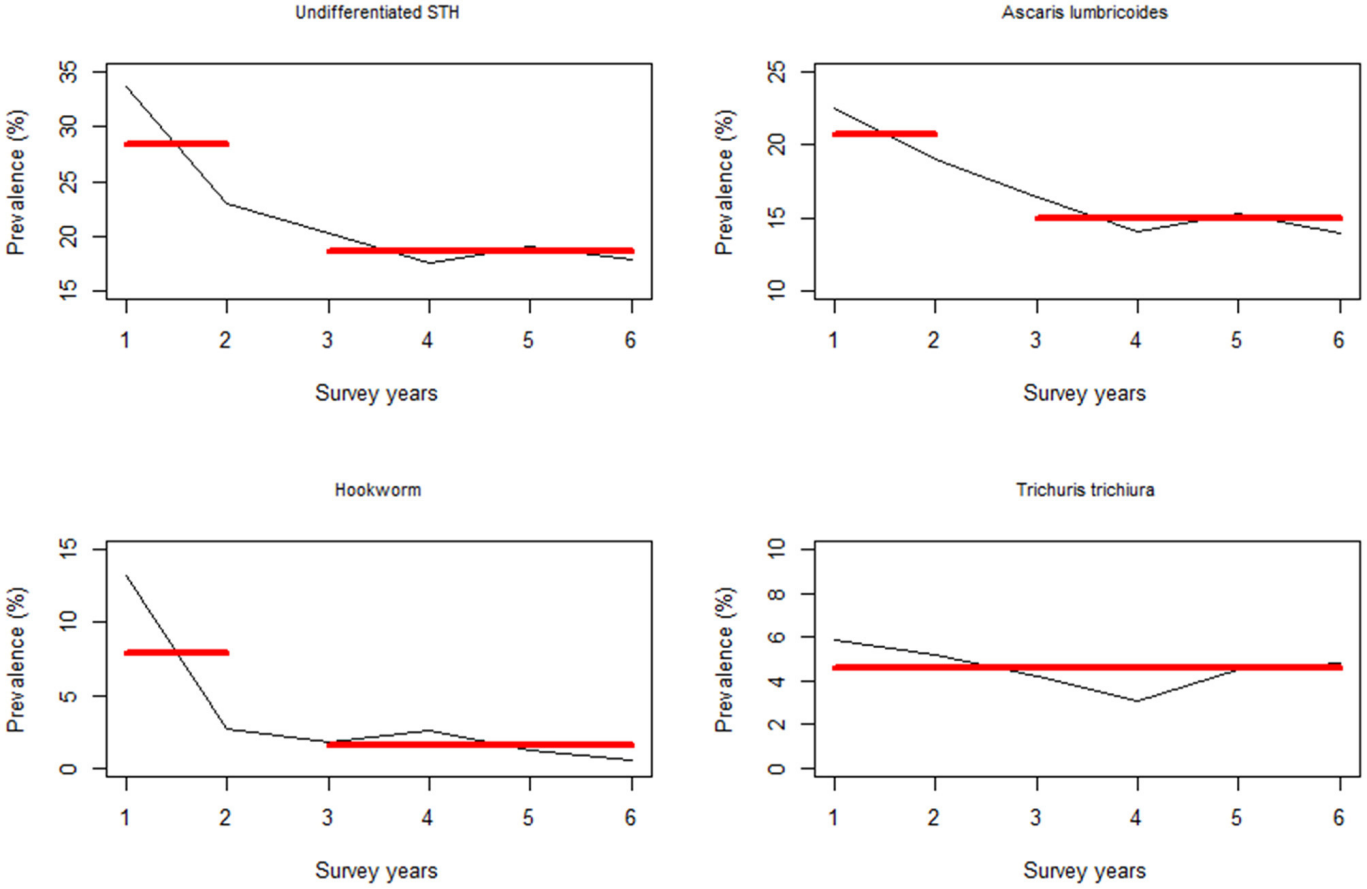
Change point analysis using binary segmentation method for the pre-treatment prevalence (%) among the preschool aged (PSAC) group of children in Kenya.

Change point analysis was performed to investigate the survey year points associated with significant changes in prevalence following the previous year(s) MDA. From this analysis, it was determined that a significant change in PSAC undifferentiated prevalence occurred during Y2 survey (i.e., after one round of MDA) for all the STH infections except *T. trichiura*, which didn't show any significant change points in prevalence (Figure 6).

**Figure 7:**
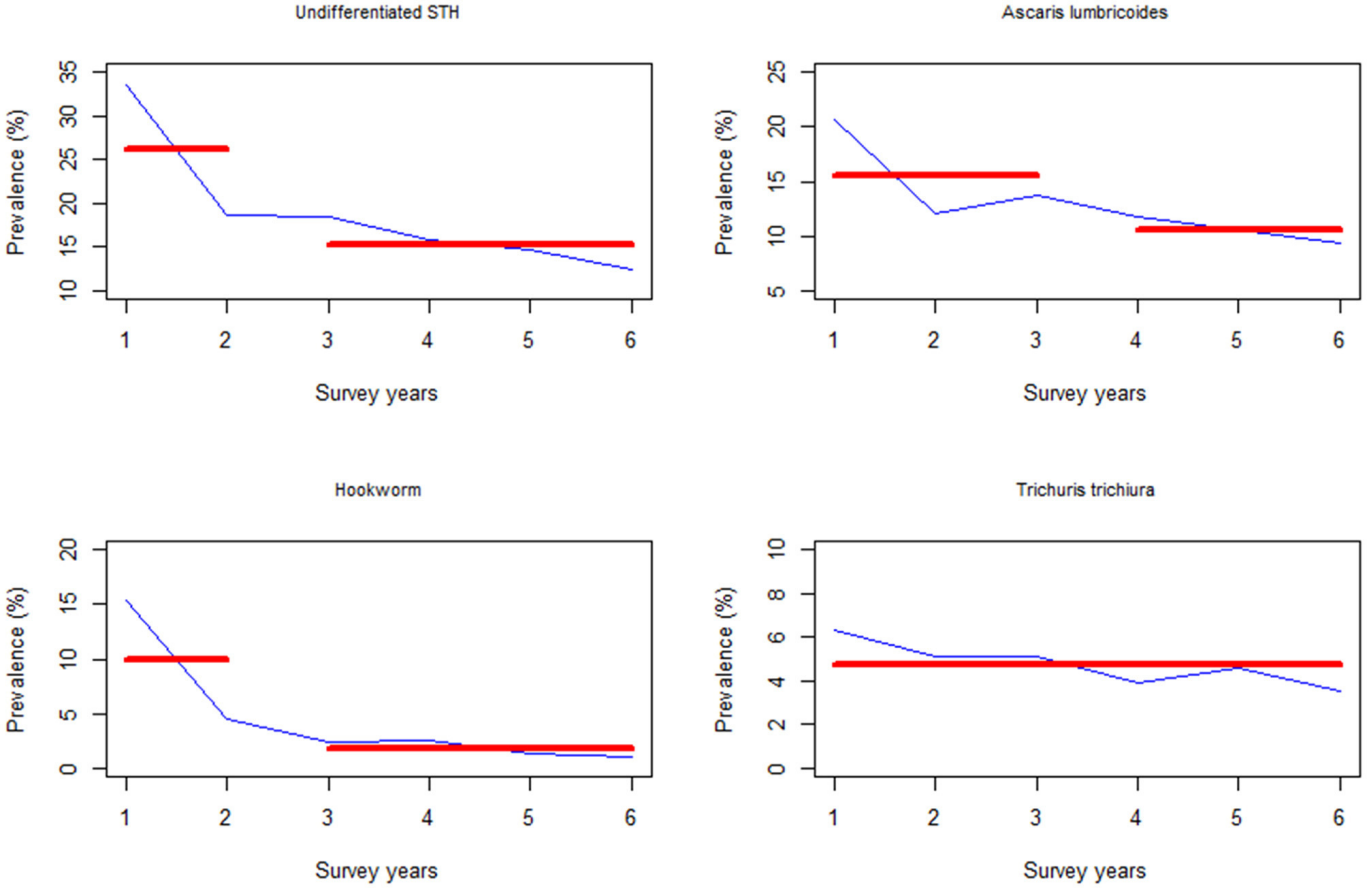
Change point analysis using binary segmentation method for the pre-treatment prevalence (%) among the school aged (SAC) group of children in Kenya.

Additionally, the change points associated with the SAC infection prevalence occurred during Y2 survey time point for undifferentiated STH and hookworm infection, while for *A. lumbricoides* it occurred during Y3 (i.e., after two rounds of MDA). Similarly, there was no significant change points associated with *T. trichiura* (Figure 7).

## Discussion

In this study, we quantified the correlation between the observed school level STH prevalence and treatment coverage, in order to understand how treatment coverage influenced observed reduction or increase in prevalence at county level. Past mathematical models have predicted that high sustained treatment coverage levels (above 75%) in low and medium transmission settings can eventually result in transmission interruption, but in high transmission areas the high coverage needs to be coupled with biannual treatment frequency and extended to include adults, in order to achieve transmission interruption (28). In addition, we provided analysis comparing the STH infections prevalence among PSAC and SAC groups of children within schools participating in M&E activities of the Kenyan NSBD program at four key evaluation survey time points following 6 years of sustained annual mass treatment. This will provide information to Kenyan STH control programs about STH burden among the two most vulnerable populations in the country. Previous studies of data from M&E activities as part of the NSBD program have focused on infection burden in the general school going children population without disaggregation between these two critical age groups (14–17).

Average treatment coverage through all six surveys was reportedly above 78%, which exceeds the WHO minimum effective treatment coverage level of 75% (19). In each age group, the yearly treatment coverage was also above 75% with the exception of PSAC during Y1 where coverage was only an average of 21.6% across counties. This was likely due to the initially low awareness of the program only offering treatment to enrolled children resulting in non-enrolled PSAC, a likely substantial proportion, being left untreated. County-level treatment coverage among each age group was heterogeneous and varied from year to year. Efforts should be made by the NSBD program to ensure that county level treatment coverage remains above the WHO recommended thresholds.

### PSAC and SAC Infection Prevalence

After 6 years of MDA and rigorous M&E, we found that 17.9% of PSAC and 12.5% of SAC were infected with at least one STH infection, noting that both groups of children had similar infection burdens just prior to the implementation of the NSBD program (Y1). Our analysis suggested a slightly higher infection burden among PSAC than SAC. The fact that PSAC children still harbor a higher burden of STH infection points to a missed opportunity for treating this vulnerable group of children by STH control programs during deworming activity. Consideration should be given to increasing the deworming coverage for PSAC.

Prevalence of differentiated STH infections differed across age structures, with *A. lumbricoides* being prevalent among PSAC while hookworm and *T. trichiura* being predominant among SAC across all surveys. This finding is in line with the current research pointing to an increase in hookworm and *T. trichiura* prevalence and intensity with age (29–31). This suggests that the NSBD program might have had less impact on the community-wide prevalence where these two infections are likely be common, especially among adults (32, 33). As suggested in previous studies, a large burden of STH infections are likely harbored in adults, underscoring the importance of potentially broadening treatment coverage to encompass the general community (28, 32).

An overall significant decline in prevalence for undifferentiated STH infections and specifically for hookworm and *A. lumbricoides* in both groups of children was observed, however, prevalence reductions for *T. trichiura* were only significant among SAC and not significant among PSAC. Previous studies have shown that single dose oral albendazole is efficacious against these two species of STH (34–36), but likely less efficacious against *T. trichiura* (34–37). Again, the observed non-significant reductions especially for *T. trichiura* could be due to the changing treatment coverage levels among each group of children, and differing county level coverage as well as considerable fluctuations in coverage targets and reported coverage within counties over time. For significant prevalence reductions to be observed for *T. trichiura*, higher treatment coverage levels may be warranted. Alternately dual drug therapy involving ivermectin co-administered with albendazole or mebendazole may be effective (38).

### Correlations Between Prevalence and Treatment Coverage for PSAC and SAC Groups

Correlational analysis suggested that prevalence reduced as treatment coverage increased or maintained at sufficient coverage level. Counterintuitively however, other analyses suggested infection prevalence increased or remained high despite high (or an increase in) treatment coverage. This suggests that increases in treatment coverage alone are not sufficient to influence reductions in infection prevalence. This finding is emphasized if a sudden increase in treatment coverage is followed a spell of consistently low coverage levels.

Correlational analyses are useful for investigating the relationship between STH prevalence and MDA coverage, however these remain non-causal. As a general rule, under the methods followed in this study, correlations of *r* < 0.7 are seen as relatively weak with too much potential for chance or confounding to be interpreted as meaningful. For this reason, even where statistically significant, weak correlations coupled with small sample sizes are particularly problematic to interpret. Insufficient sample sizes especially at the county level were a major barrier in this study. In the analyses that were conducted, the “overall” (all counties, all time points) correlations remain most reliable for inferring potential relationships, largely due to larger sample sizes.

Overall, across all survey time points, significant negative correlations between infection prevalence and treatment coverage were observed for undifferentiated STH infections among PSAC, however not for SAC. This suggests that treatment coverage directly affects reductions in PSAC prevalence. This finding however, may be influenced by relatively few PSAC being included in the M&E surveys, compared to their SAC counterparts.

County level correlations were observed between increased coverage in MDA and reductions in prevalence; for both PSAC and SAC in Bomet, Bungoma, Kakamega, and Vihiga counties; for PSAC only in Kilifi, Kwale, Mombasa, Kisumu, and Migori; and for SAC only in Kisii and Taita Taveta. The observed significant negative correlations in the coastal counties of Kilifi, Kwale, Mombasa, and Taita Taveta may be attributed to additional community-wide treatments provided by other partner programs that used albendazole. For instance, the Kenyan *Lymphatic filariasis* Elimination Program has annually provided, on average, 10 rounds of antifilarial tablets; diethylcarbamazine citrate (DEC) and albendazole to the entire at-risk populations aged 2 years and above for community-wide treatment and control of *Lymphatic filariasis* in six counties of the coastal region since 2002 (39). Similarly, observed significant negative correlations in western region counties, especially Bungoma, Kakamega, and Vihiga, may be attributed to supplementary treatment, and revamped water, sanitation and hygiene (WASH) interventions by the *WASH Benefits Study* which provided interventions to households in 702 villages between November 2012 and May 2014 (40). These examples of additional treatment by partner programs illustrate that the provision of school-based deworming coupled with community based treatment, as well as contemporary interventions like WASH, may lead to achievement of significant reductions in prevalence.

Statistically significant correlations between increased coverage, and also increases in prevalence were mostly observed during Y6 survey for both PSAC and SAC for undifferentiated STH and specifically for *A. lumbricoides*. The positive correlations observed during this particularly survey year can be mainly attributed to the fact that many areas had received treatment below 75% during the past immediate MDA round compared to the preceding MDAs. Also, prevalence across many schools were still high at above 20%. County level significant positive correlations were observed in few counties such as Migori, Homabay, and Kisumu. Most of these counties had histories of limited additional interventions from partner programs and generally relied solely on school-based deworming as a mainstay control measure. This points to the fact that school deworming alone is not sufficient to provide high treatment coverage necessary to maintain sustained reductions in prevalence over long time periods.

The absence of a more pronounced relationship between increasing/maintained coverage and prevalence decreases may be explained by the non-linear relationship between infection prevalence and treatment coverage as outlined in Figure 5. What we see from this figure is that treatment coverage likely needs to be maintained above a critical minimum level in order to achieve a sustained reduction in prevalence. We note that, minimum coverage level varies by survey time point as well as individual STH species. However, for the possible elimination of STH as a public health problem by treatment alone, minimum treatment coverage needs to be maintained above 85%. We appreciate that, our findings differ from the WHO recommended minimum coverage level of 75% (19). However, this is expected as infection levels in Kenya have substantially reduced since the inception of the NSBD program and therefore higher treatment coverage levels are necessary to “mob up” remaining prevalence.

Among PSAC, low coverage coupled with small sample sizes especially at county level, essentially resulted in a lack of statistical power to conduct correlation analysis at county-level and likely contributed to variability across results. It should further be noted that the prevalence surveys used in this analysis were not designed for county-level analyses, and are themselves insufficiently powered at the county level for strong conclusions to be drawn. As county level treatment coverage among PSAC was well-below 75% at most time points, and the proportion of PSAC at each time point represented treatment-naïve children who would have only just become old enough to start receiving treatments, it is not possible to conclude that a lack of reduction in prevalence is due to resurgence of infection between MDA rounds. However, these results do indicate that there are either considerable numbers of untreated PSAC and/or that there is high transmission occurring from the environment in which these children live.

The emergence of very few strong correlations at county levels could be possibly due to variability between levels of treatment coverage and infection prevalence across different counties year-on-year, accompanied by generally low numbers both for coverage and prevalence at county level, negating the opportunities to find correlations both within and across time points. Lack of statistical power at county level, notwithstanding, this analysis has enabled us to identify areas which could benefit from more focused activities like detailed qualitative research in counties like Homabay, Kisumu, Migori, and other counties which showed positive correlations, either overall or at specific time points, especially among SAC. Such research would enable a greater understanding of why prevalence continues to rise despite relatively high coverage levels.

### Change Point Analysis

Drawing from our change point analysis, there was a significant change in undifferentiated STH prevalence, specifically hookworm, after one round of MDA (where the change point occurred at Y2) in both groups of children. However, hookworm prevalence in subsequent years (i.e., after Y2) did not vary sufficiently to warrant detection of any change. This finding on hookworm suggests the high efficacy of treatment using albendazole on both PSAC and SAC, which has been widely demonstrated in other studies (34–36).

Change points associated with *A. lumbricoides* were observed during Y2 (for PSAC) and Y3 (for SAC). Again this finding suggests that albendazole is highly efficacious against this particular infection (34–36), and that the NSBD program has been able to significantly reduce the parasites prevalence after only 2 to 3 rounds of MDA in both groups. It is worthwhile to note that change point in the SAC group took a little longer to be realized and this could be as a result of the high levels of re-infections that are often associated with *A. lumbricoides* (37). Going forward, the NSBD program should take measures to address the issue of *A. lumbricoides* re-infections.

Finally, we did not observe any change points associated with *T. trichiura* infection (i.e., no detectable significant changes in prevalence over the years). As described above, the use of albendazole as the main anthelminthic drug by the NSBD program would unlikely lead to any significant reduction in *T. trichiura*. For *T. trichiura* to be eliminated as a public health problem, a drug combination needs to be used (38).

### Study Strengths and Limitations

The main strength of this study was the ability to gather 6-year data from a large scale deworming programme in Kenya. It is our view the results presented in this study will be important in promoting MDA throughout Kenya, especially the age-differentiated MDA.

However, this study was not without limitations: (I) The schools surveyed by the M&E program since 2012 were sampled and statistically powered to detect changes in infection prevalence at the national and regional levels only. Introduction of devolution in 2013 after the start of the program, yielded the county system essentially by merging and/or splitting the existing districts. Hence, at this stage, county level analysis becomes insufficiently powered to give firm conclusions at county level due to the resulting small sample sizes and the inconsistent number of schools. (II) There were relatively few PSAC treated as well as examined for STH infections at each school location, this is because this group of children were not the main target of the program and were not necessarily enrolled in the participating schools and the program did not offer treatment or even conduct sample collection at household level, where they reside, rather these were done at school level. Hence, PSAC included in this study were majority drawn from children attending ECD classes as well as those who happen to be present in school during either treatment or sample collection. (III) The program used the Kato-Katz technique for examination of STH eggs in line with the WHO guidelines for examination of these infections in high endemic settings (18), however, this technique has previously been shown to be less sensitive in low endemic areas (41), which is particularly salient during years 5 and 6 in which prevalence reduced substantially. Therefore, the prevalence estimates presented here may be an under-estimation of the true population prevalence. (IV) Finally, it is important to note that the NSBD program currently only follows schools longitudinally and not individual children.

## Conclusions

In this article, we have provided detailed information on PSAC and SAC infection prevalence, treatment coverage, relative reductions in prevalence, and correlation analysis between treatment coverage and infection prevalence. Our findings showed an initially higher burden of STH infections among both groups of children which declined steadily over the survey years. Significant negative correlations in some counties indicated that prevalence reduced over time as treatment coverage increased or remained at a sufficiently high level as previously determined by WHO. The absence of many strong significant negative correlations might be attributed to several factors including low baseline infection prevalence and/or low treatment coverage preventing elucidation of correlations, or other factors, not measured in this analysis, such as an insufficient number of treatment rounds, reinfections, insufficient WASH access, and/or reduced drug efficacy among other possible factors. Finally, for the first time in Kenya, the overlay of treatment coverage and prevalence data indicated that there are minimum treatment coverage thresholds needed to achieve a negative relationship ensuring that prevalence will decline as coverage increases, this imply that administration of MDA may be most impactful if applied at county level and maintained above key thresholds.

## Data Availability Statement

The original contributions generated for the study are included in the article/Supplementary Material, further inquiries can be directed to the corresponding author/s.

## Ethics Statement

The studies involving human participants were reviewed and approved by Kenya Medical Research Institute (KEMRI)'s Scientific and Ethics Review Unit (SSC Number 2206). Written informed consent to participate in this study was provided by the participants' legal guardian/next of kin.

## Consent to Participate

Ethical approval for the original M&E study protocol was obtained from the Kenya Medical Research Institute (KEMRI)'s Scientific and Ethics Review Unit (SSC Number 2206). At county-level, approval was provided by the respective county health and education authorities. At school, written informed consent to participate in this study was provided by the participants' legal guardian/next of kin. Additionally, individual assent was obtained from each child before participation in the study. Further, the datasets for this additional analysis were requested from and approved by the KEMRI's M&E program and Evidence Action. All data used were anonymised.

## Author Contributions

COk participated in the study design, data curation and analysis, developed the initial draft manuscript. SJC, MM, and COw participated in the study design, data curation, and interpretation of the findings. NO and GM provided additional review of the study design and interpretation of the findings. CM provided overall scientific guidance during the study design, analysis, and writing of the manuscript. All authors participated in the interpretation of the findings, read and approved the final manuscript.

## Conflict of Interest

The authors declare that the research was conducted in the absence of any commercial or financial relationships that could be construed as a potential conflict of interest.

## Abbreviations

CIs: Confidence intervals
DEC: Diethylcarbamazine citrate
ECD: Early childhood development
MDA: Mass drug administration
M&E: Monitoring and evaluation
NSBD: National school based deworming
NTDs: Neglected tropical diseases
PSAC: Preschool aged children
RR: Relative reduction
SAC: School aged children
SEM: Structural equation model
STH: Soil-transmitted helminths
WASH: Water sanitation and hygiene
WCA: Women of childbearing age
WHO: World Health Organization
Y1: Year 1
Y3: Year 3
Y5: Year 5
Y6: Year 6.

## Appendix

**A1**. The statistical correlations between infection prevalence and the previous year's treatment coverage were fitted using a structural equation model (SEM) of the following structure (Figure A1):
