# Supplementary material for: Prevalence and Correlation Analysis of Soil-Transmitted Helminths Infections and Treatment Coverage for Preschool and School Aged Children in Kenya: Secondary Analysis of the National School Based Deworming Program Data
Source: Front Public Health. 2021 Jul 16;9:645522. doi: 10.3389/fpubh.2021.645522 (PMC8322119; doi:10.3389/fpubh.2021.645522)
Supplement: Supplementary file 1 [file Table_1.DOCX]

**Supplementary file**

**Table S1: Specific survey time points correlations between pre-treatment differentiated STH prevalence of infection and previous year treatment coverage by county among pre-school (PSAC) and school (SAC) aged group of children in Kenya**

| **County/**  **Survey^$^** | **Year 3 [Correlation(r), p-value]** | | | **Year 5 [Correlation(r), p-value]** | | | **Year 6 [Correlation(r), p-value]** | | |
| --- | --- | --- | --- | --- | --- | --- | --- | --- | --- |
|  | ***A. lumbricoides*** | **hookworm** | ***T. trichiura*** | ***A. lumbricoides*** | **hookworm** | ***T. trichiura*** | ***A. lumbricoides*** | **hookworm** | ***T. trichiura*** |
| **PSAC** |  |  |  |  |  |  |  |  |  |
| Bomet | r=-0.664, p<0.001* | Insufficient obs | r=0.142, p=0.681 | r=-0.070, p=0.802 | Insufficient obs | r=-0.316, p=0.297 | r=0.503, p=0.045^#^ | Insufficient obs | Insufficient obs |
| Bungoma | r=-0.171, p=0.594 | r=-0.333, p=0.066 | Insufficient obs | r=0.121, p=0.716 | r=-0.333, p=0.066 | Insufficient obs | r=-0.604, p=0.014* | r=-0.408, p=0.110 | Insufficient obs |
| Busia | r=0.471, p=0.010^#^ | r=-0.326, p=0.048* | r=-0.082, p=0.609 | r=-0.157, p=0.099 | r=0.174, p=0.567 | r=-0.189, p=0.062 | Insufficient obs | Insufficient obs | Insufficient obs |
| Garissa | -^ns^ | -^ns^ | -^ns^ | -^ns^ | -^ns^ | -^ns^ | Insufficient obs | Insufficient obs | Insufficient obs |
| Homa Bay | r=0.158, p=0.325 | r=0.259, p=0.082 | r=0.200, p=0.020^#^ | r=-0.041, p=0.861 | r=-0.312, p=0.006* | r=-0.227, p=0.054 | Insufficient obs | Insufficient obs | Insufficient obs |
| Kakamega | r=0.516, p<0.001^#^ | Insufficient obs | r=0.271, p=0.050 | r=-0.376, p=0.030* | r=-0.224, p=0.049* | Insufficient obs | r=0.196, p=0.600 | r=0.408, p=0.110 | r=0.408, p=0.110 |
| Kericho | r=0.233, p=0.530 | r=0.294, p=0.123 | r=-0.400, p=0.016* | r=-0.182, p=0.514 | Insufficient obs | r=0.198, p=0.474 | r=0.450, p=0.028^#^ | Insufficient obs | r=-0.250, p=0.191 |
| Kilifi | Insufficient obs | Insufficient obs | Insufficient obs | Insufficient obs | Insufficient obs | Insufficient obs | Insufficient obs | Insufficient obs | Insufficient obs |
| Kisii | r=0.152, p=0.684 | Insufficient obs | Insufficient obs | r=-0.545, p=0.028* | r=-0.085, p=0.407 | r=-0.504, p=0.014* | r=-0.625, p=0.002* | Insufficient obs | **r=-0.824, p<0.001*°** |
| Kisumu | Insufficient obs | r=0.218, p=0.092 | r=0.218, p=0.092 | r=0.316, p=0.075 | Insufficient obs | r=0.141, p=0.623 | Insufficient obs | Insufficient obs | r=0.086, p=0.792 |
| Kitui | -^ns^ | -^ns^ | -^ns^ | -^ns^ | -^ns^ | -^ns^ | Insufficient obs | Insufficient obs | Insufficient obs |
| Kwale | Insufficient obs | r=0.064, p=0.795 | r=-0.112, p=0.475 | Insufficient obs | r=-0.038, p=0.913 | r=0.203, p=0.480 | Insufficient obs | Insufficient obs | Insufficient obs |
| Makueni | -^ns^ | -^ns^ | -^ns^ | -^ns^ | -^ns^ | -^ns^ | Insufficient obs | Insufficient obs | Insufficient obs |
| Migori | Insufficient obs | Insufficient obs | Insufficient obs | Insufficient obs | Insufficient obs | Insufficient obs | Insufficient obs | Insufficient obs | Insufficient obs |
| Mombasa | Insufficient obs | Insufficient obs | Insufficient obs | Insufficient obs | Insufficient obs | Insufficient obs | Insufficient obs | Insufficient obs | r=-0.250, p=0.191 |
| Narok | r=-0.219, p=0.114 | Insufficient obs | r=-0.459, r=0.041* | r=-0.135, p=0.604 | r=-0.189, p=0.117 | r=-0.597, p=0.001* | Insufficient obs | Insufficient obs | Insufficient obs |
| Nyamira | r=-0.326, p=0.047* | Insufficient obs | Insufficient obs | r=-0.342, p=0.034* | Insufficient obs | Insufficient obs | r=0.222, p=0.552 | Insufficient obs | Insufficient obs |
| T. Taveta | Insufficient obs | Insufficient obs | Insufficient obs | Insufficient obs | Insufficient obs | Insufficient obs | Insufficient obs | Insufficient obs | Insufficient obs |
| Vihiga | r=-0.614, p=0.002* | Insufficient obs | r=-0.378, p=0.071 | r=-0.669, p<0.001* | r=-0.343, p=0.269 | r=-0.248, p=463 | r=-0.236, p=0.669 | Insufficient obs | r=0.408, p=0.110 |
| Wajir | -^ns^ | -^ns^ | -^ns^ | -^ns^ | -^ns^ | -^ns^ | Insufficient obs | Insufficient obs | Insufficient obs |
| **Overall** | **r=0.076, p=0.305** | **r=0.004, p=0.958** | **r=0.031, p=0.665** | **r=-0.109, p=0.237** | **r=-0.127, p=0.069** | **r=0.029, p=0.654** | **r=0.325, p<0.001^#^** | **r=0.059, p=0.111** | **r=-0.044, p=0.571** |
| **SAC** |  |  |  |  |  |  |  |  |  |
| Bomet | **r=-0.727, p<0.001*°** | r=0.330, p=0.054 | r=-0.298, p=0.310 | r=-0.670, p<0.001* | r=0.118, p=0.260 | r=-0.599, p=0.006* | r=-0.255, p=0.424 | Insufficient obs | r=0.379, p=0.104 |
| Bungoma | r=-0.325, p=0.306 | r=-0.277, p=0.392 | Insufficient obs | r=-0.152, p=0.642 | r=-0.056, p=0.852 | r=-0.102, p=0.763 | r=0.187, p=0.734 | Insufficient obs | Insufficient obs |
| Busia | r=0.129, p=0.551 | r=0.456, p=0.005^#^ | r=-0.191, p=0.363 | r=-0.133, p=0.587 | r=0.692, p<0.001^#^ | r=-0.650, p<0.001* | Insufficient obs | Insufficient obs | Insufficient obs |
| Garissa | -^ns^ | -^ns^ | -^ns^ | -^ns^ | -^ns^ | -^ns^ | Insufficient obs | Insufficient obs | Insufficient obs |
| Homa Bay | r=-0.282, p=0.146 | r=0.087, p=0.655 | r=-0.182, p=0.403 | r=-0.141, p=0.489 | r=0.151, p=0.396 | r=-0.022, p=0.921 | r=-0.423, p=0.323 | r=-0.182, p=0.734 | **r=-0.903, p<0.002*°** |
| Kakamega | r=0.015, p=0.953 | r=0.076, p=0.721 | r=-0.305, p=0.056 | r=-0.527, p<0.001* | r=-0.431, p<0.001* | r=-0.458, p<0.001* | r=-0.374, p=0.417 | r=-0.461, p=0.048* | r=0.408, p=0.110 |
| Kericho | r=-0.278, p=0.245 | Insufficient obs | r=-0.100, p=0.711 | r=-0.207, p=0.397 | r=0.495, p=0.009^#^ | r=-0.136, p=0.620 | r=-0.675, p=0.002* | r=-0.250, p=0.191 | r=-0.129, p=0.628 |
| Kilifi | Insufficient obs | Insufficient obs | Insufficient obs | Insufficient obs | Insufficient obs | Insufficient obs | r=-0.458, p=0.052 | r=-0.273, p=0.270 | r=-0.156, p=0.455 |
| Kisii | r=-0.255, p=0.136 | r=-0.296, p=0.179 | **r=-0.708, p=0.003*°** | r=-0.138, p=0.660 | r=0.556, p=0.014^#^ | r=-0.198, p=0.365 | r=0.495, p=0.055 | r=0.250, p=0.191 | r=-0.537, p=0.049* |
| Kisumu | **r=0.721, p<0.001^#^°** | r=0.676, p<0.001^#^ | r=0.564, p=0.003^#^ | r=-0.608, p=0.013* | r=-0.418, p=0.161 | r=-0.087, p=0.759 | r=-0.385, p=0.132 | r=-0.017, p=0.945 | r=0.273, p=0.291 |
| Kitui | -^ns^ | -^ns^ | -^ns^ | -^ns^ | -^ns^ | -^ns^ | Insufficient obs | Insufficient obs | Insufficient obs |
| Kwale | r=-0.187, p=0.346 | r=-0.626, p=0.003* | **r=-0.967, p<0.001*°** | Insufficient obs | r=0.497, p=0.010^#^ | **r=0.798, p<0.001^#^°** | Insufficient obs | Insufficient obs | Insufficient obs |
| Makueni | -^ns^ | -^ns^ | -^ns^ | -^ns^ | -^ns^ | -^ns^ | Insufficient obs | Insufficient obs | Insufficient obs |
| Migori | Insufficient obs | Insufficient obs | Insufficient obs | Insufficient obs | Insufficient obs | Insufficient obs | Insufficient obs | Insufficient obs | Insufficient obs |
| Mombasa | Insufficient obs | r=-0.100, p=0.001* | r=-0.255, p=0.635 | Insufficient obs | Insufficient obs | r=0.500, p=0.182 | r=0.056, p=0.821 | r=0.056, p=0.821 | r=-0.680, p=0.001* |
| Narok | r=-0.043, p=0.836 | r=-0.016, p=0.962 | r=-0.637, p<0.001* | r=0.254, p=0.377 | r=-0.421, p=0.008* | **r=-0.706, p<0.001*°** | Insufficient obs | Insufficient obs | Insufficient obs |
| Nyamira | r=0.240, p=0.098 | r=0.355, p=0.079 | r=-0.333, p=0.066 | r=0.070, p=0.545 | r=-0.111, p=0.177 | **r=0.946, p<0.001^#^°** | r=0.422, p=0.108 | r=-0.250, p=0.191 | r=0.066, p=0.807 |
| T. Taveta | Insufficient obs | Insufficient obs | Insufficient obs | Insufficient obs | Insufficient obs | Insufficient obs | **r=-0.980, p<0.001*°** | Insufficient obs | Insufficient obs |
| Vihiga | r=-0.613, p=0.029* | r=0.064, p=0.863 | r=-0.617, p<0.001* | r=-0.645, p=0.004* | r=-0.179, p=0.576 | r=-0.451, p=0.015* | r=-0.313, p=0.467 | r=-0.670, p<0.001* | r=-0.355, p=0.328 |
| Wajir | -^ns^ | -^ns^ | -^ns^ | -^ns^ | -^ns^ | -^ns^ | Insufficient obs | Insufficient obs | Insufficient obs |
| **Overall** | **r=-0.191, p=0.055** | **r=-0.026, p=0.669** | **r=-0.015, p=0.764** | **r=-0.238, p=0.019*** | **r=-0.013, p=0.858** | **r=-0.002, p=0.977** | **r=0.370, p<0.001^#^** | **r=-0.159, p=0.179** | **r=0.095, p=0.023^#^** |
| ^$^Year 1 correlations were not included since there was no previous treatment coverage to compare with the Year 1 prevalence.  -^ns^ Indicates counties which had not had routine parasitological monitoring, hence, surveys were not conducted in these counties during Year 1, Year 3 and Year 5 assessments.  *Indicates a statistically significant negative correlation.  ^#^Indicates a statistically significant positive correlation.  °Indicates a strong significant correlation (defined as r>0.7, p<0.001). | | | | | | | | | |
